# Limiting factors for wearing personal protective equipment (PPE) in a health care environment evaluated in a randomised study

**DOI:** 10.1371/journal.pone.0210775

**Published:** 2019-01-22

**Authors:** Martina Loibner, Sandra Hagauer, Gerold Schwantzer, Andrea Berghold, Kurt Zatloukal

**Affiliations:** 1 Medical University Graz, Institute of Pathology, Christian Doppler Laboratory for Biospecimen Research and Biobanking Technologies, Graz, Austria; 2 Medical University Graz, Institute for Medical Informatics, Statistics and Documentation, Graz, Austria; Brigham and Women’s Hospital, Harvard Medical School, UNITED STATES

## Abstract

Pandemics and re-emerging diseases put pressure on the health care system to prepare for patient care and sample logistics requiring enhanced personnel protective equipment (PPE) for health care workers. We generated quantifiable data on ergonomics of PPE applicable in a health care setting by defining error rates and physically limiting factors due to PPE-induced restrictions. Nineteen study volunteers tested randomly allocated head- or full body-ventilated PPE suits equipped with powered-air-purifying-respirators and performed four different tasks (two laboratory tutorials, a timed test of selective attention and a test investigating reaction time, mobility, speed and physical exercise) during 6 working hours at 22°C on one day and 4 working hours at 28°C on another day. Error rates and physical parameters (fluid loss, body temperature, heart rate) were determined and ergonomic-related parameters were assessed hourly using assessment sheets. Depending on the PPE system the most restrictive factors, which however had no negative impact on performance (speed and error rate), were: reduced dexterity due to multiple glove layers, impaired visibility by flexible face shields and back pain related to the respirator of the fully ventilated suit. Heat stress and liquid loss were perceived as restrictive at a working temperature of 28°C but not 22°C.

## Introduction

Pandemics and re-emerging diseases put pressure on the health care system to prepare for patient care and sample management for diagnostics requiring personnel protective equipment (PPE) for exposed health care workers (HCWs). The majority of European hospitals are not equipped with isolation units for patients and high security containments for sample management in the event of emerging or re-emerging infectious diseases with high risk potential. Specific challenges in a hospital environment are patient care, the handling of infectious samples for diagnostics and the work with dead bodies.

Various protective suit systems for different applications are commercially available and the selection of the most appropriate PPE (optimal protection, best ergonomic features and best tolerance by wearers) should be based on evidence. Performing hazardous laboratory or clinical work while wearing PPE involves various constrictions compared to the same work without PPE. For example, multiple layers of gloves limit dexterity, long-term work in PPE may cause heat stress, viewing foils of face shields (dependent on the suit system) reflect and refract the light making observation stressful and leading to eye fatigue. Therefore, health care professionals wearing chemical, biological, radiation and nuclear personal protective equipment (CBRN-PPE) and performing intubation and intravenous cannulation are significantly slower or even unsuccessful compared to personnel with the same skills performing the same work under standard conditions as shown by a UK study mimicking time critical emergency casualties caused by a CBRN incident [1]. Apart from the information provided by the suit manufacturers, not many systematic studies exist on the requirements of CBRN-PPE, particularly for HCWs. Consequently, there is a need to test various types of PPE for material compatibility, determination of protection factors, wearer comfort and additional adaptations to specific needs [2, 3]. So far, tests mimicking the event of a biological threat have been performed in different contexts, for example, the assessment of whole hospitals by the French “Biotox-Pirotox” Network [4], performance of resuscitation skills wearing PPE [5, 6], and evaluation of PPE protection factors [7, 8]. Furthermore, valuable experience was obtained from performing autopsies in BSL3 facilities of cases with severe acute respiratory syndrome coronavirus (SARS-CoV) in China [9].

Increased awareness of the impact and need for measures to protect HCWs and the population from biological threats has been triggered by infections with Crimean-Congo hemorrhagic fever virus (CCHFV) [10] and the quick spread of SARS-CoV originating from the Guangdong Province of China to 33 countries worldwide within 6 months [9]. The Ebola outbreak in West Africa demonstrated the need for a quick response to unusual emerging infections using rapidly deployable field laboratory equipment and that wearing of PPE has several limitations under these conditions [11, 12, 13].

Recent studies highlight that additional research and comparative studies on various types of PPE are needed to determine optimal PPE for HCWs [14, 15]. Furthermore, testing of current PPE configurations under simulated environmental conditions to determine the length of time they could be safely worn is recommended [14]. To identify parameters affecting the performance and tolerability of wearing PPE we have investigated in a pilot study format how wearing PPE influences physical performance, individual wellbeing, concentration and error rates (wrongly processed items in different tasks) by performing series of different tasks simulating typical working steps of handling infectious materials in a health care environment under normal (22°C) and increased (28°C) working temperatures. In particular, we evaluated advantages and disadvantages of two different PPE systems and investigated which of the above mentioned parameters were most limiting for working under the tested conditions.

## Materials and methods

In this study following good clinical practice guidelines nineteen study volunteers were recruited to wear one of two different types of randomly allocated PPE suits using the web-based randomizer software (www.randomizer.at) by the Institute for Medical Informatics, Statistics and Documentation of the Medical University Graz (IMI). The software’s GCP-compliance (Good Clinical Practice) has been confirmed by the Austrian Agency for Health and Food Safety. Subjects had to perform four different tasks six times at 22°C on one day and four times at 28°C on another day in the local core facility clinical research center. Recruitment and data collection was done between May and July 2011. The study was registered at ClinicalTrials.gov (NCT03004690, “Testing of Personal Protective Equipment (PPE)) after its completion since the aim was not to provide data for a certification or approval process for medical products or devices.

### Study participants

Recruiting of healthy participants was based on voluntary registration in response to a public announcement. The study was approved by the Ethical Committee of the Medical University Graz, Austria (No. 23–321 ex 10/11) and all persons gave written informed consent according to the Helsinki Declaration (S1–S5) Files. Key lifestyle and medical parameters were documented for every subject. Exclusion criteria were pregnancy, latex and polyvinyl chloride allergy, claustrophobia, hypotension, history of vein thrombosis, chronic obstructive pulmonary disease, epilepsy, cardiovascular and pulmonary diseases, and infectious diseases (S1 Table). Ten male and nine female volunteers were recruited, aged between 21 and 38 years with body-mass-indices from 17.3 to 32.5 (Table 1).

**Table 1:** Baseline participant´s data.

	Subjects	Male (m)	Female (f)	Mean Age (SD)	Mean Body Mass Index (SD)
Suit A	10	6	4	26.5 (5.2)	22.5 (4.3)
Suit B	9	4	5	25.2 (3.8)	21.4 (2.5)

**Fig 1:**
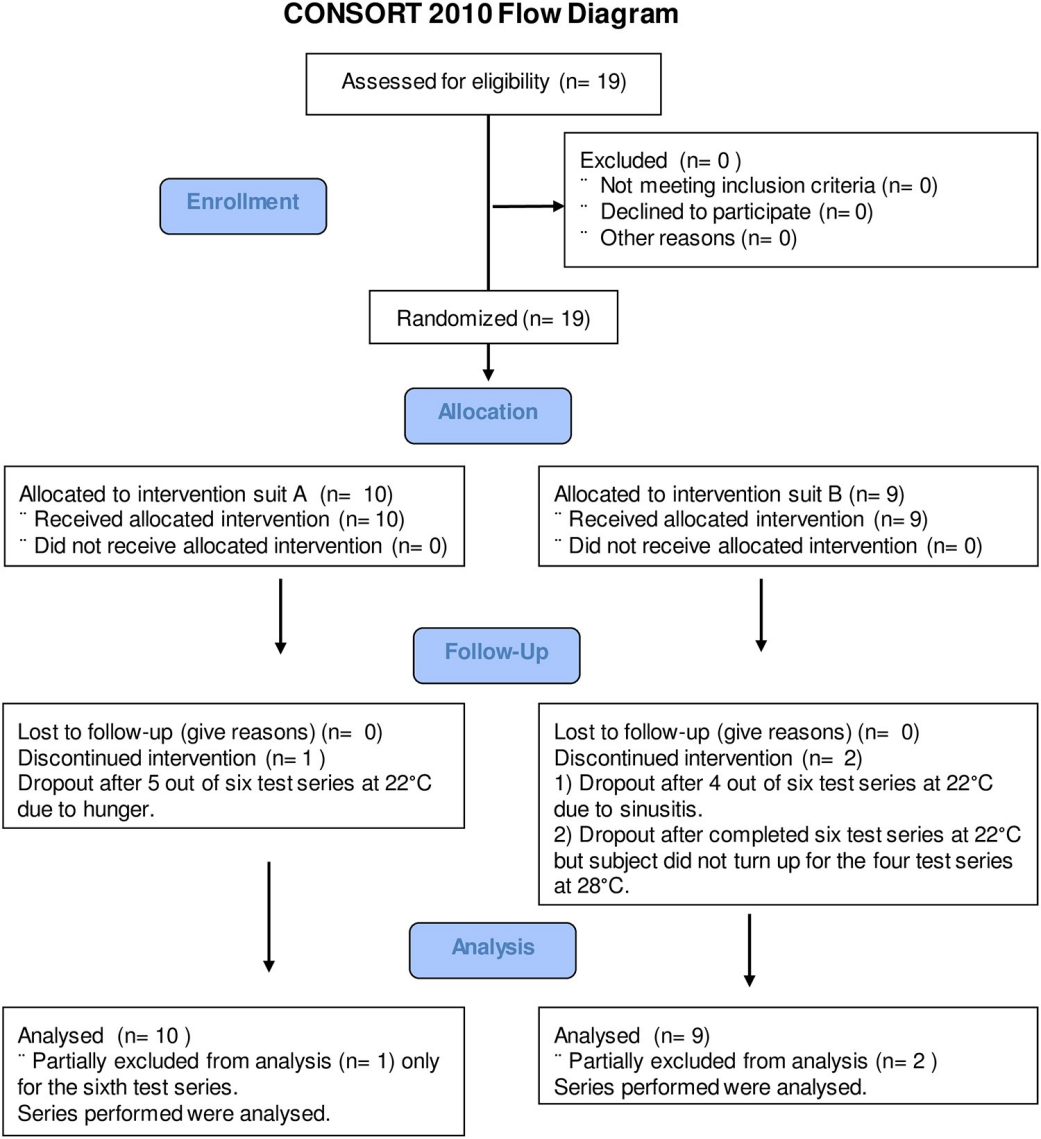
CONSORT flow diagram.

Enrollment, allocation, follow-up and analysis were performed according to the dedicated CONSORT Flow Diagram (Fig 1).

### PPE suits

**Fig 2:**
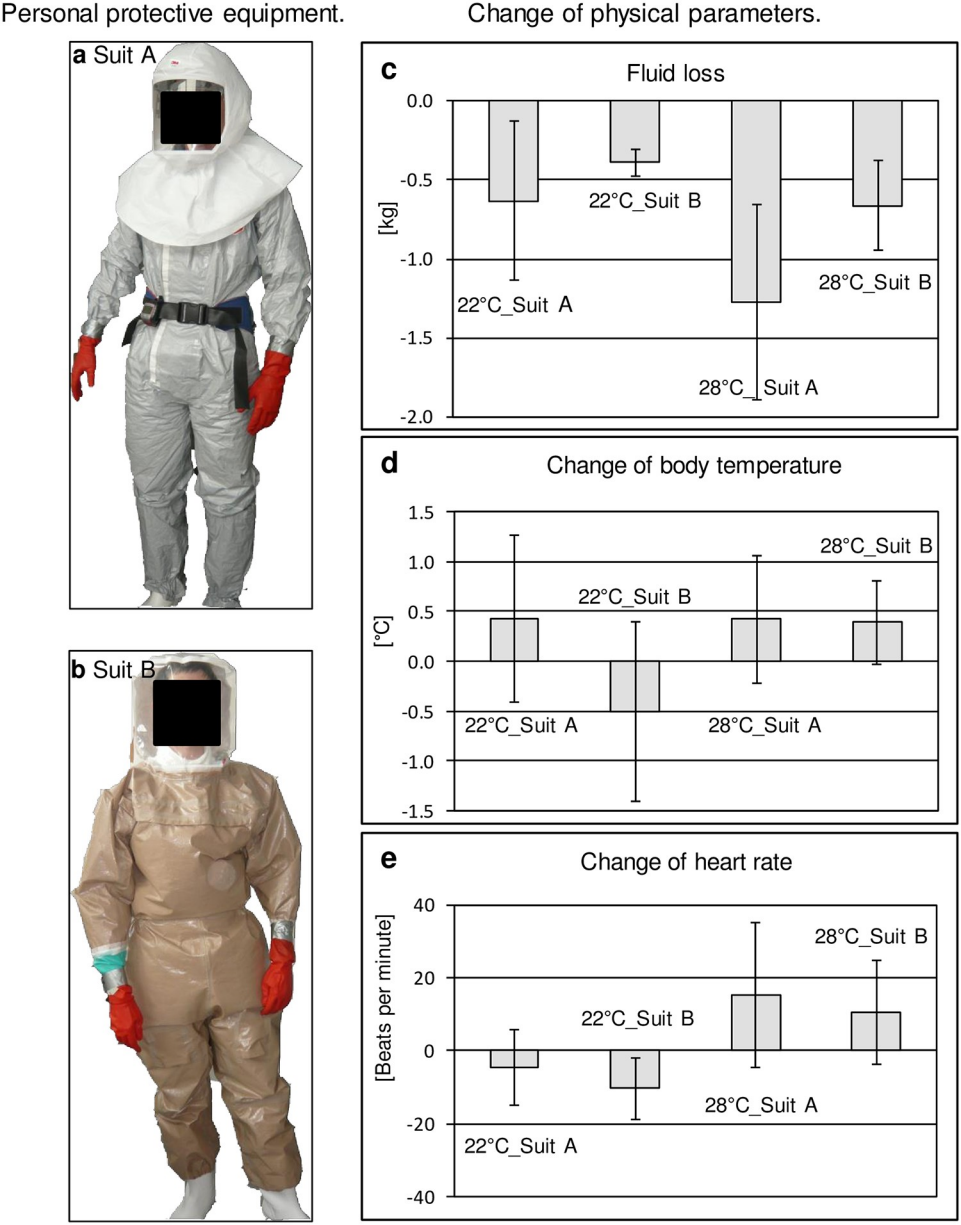
Personal protective equipment (suit A, suit B) and change of physical parameters. Suit A. (b) Suit B. (c) Fluid loss, (d) change of body temperature and (e) change of heart rate of subjects wearing different PPE were measured before the start and immediately after termination of the task series at 22°C (6 hours) and 28°C (4 hours). Mean values plus/minus standard deviation.

Suit A: TychemR F overall whole-body (DuPont de Nemours and Company, 3M, Austria) suit including socks with a reusable light hood Versaflo S-655 (3M, Austria) and an external 3M Jupiter Powered Air Turbo Unit (3M Austria) providing head-only positive pressure. Ten participants were randomly allocated to wear suit A (Fig 2a).

Suit B: 3M JS-series Typ 3 Chemical and Respiratory Protective Suit (CRPS, 3M, Austria) with integrated respirator 3M Jupiter JP-ER-03 Powered Air Purifying Turbo (3M, Austria) fixed as a rucksack generating whole-suit positive pressure. Nine participants were randomly allocated to wear suit B (Fig 2b).

With both suits, Sempercare surgical gloves (Sempermed, powder-free 150; Semperit, Austria) as the first layer, Ansell Sol-Vex gloves 37–900 (Ansell, Medical GBU, VWR, Austria) as a second layer and white rubber boots were worn. Gloves and boots were sealed to the suit using adhesive tape. Tasks I and II (Table 1a) were performed in a mock-up glove box providing a third layer of latex gloves.

### Tasks

Four tasks were repeatedly performed as well as an additional assessment on comfort and general condition before the tasks started and after every four-task series (Table 2). Task I comprised correct assembly and position of coloured and numbered 2 mL tubes and screwcaps in a storage device to test fine motor skills, concentration and error rate. Task II checked the same skills in a different approach by pipetting different volumes of coloured water into a 96-well microliter plate according to a given pattern. Task III “d2 Test of Attention” is a timed test of selective attention and a standardized refinement of a visual cancellation [16]. In response to the discrimination of similar visual stimuli, the test measures processing speed, rule compliance, and quality of performance, allowing estimation of individual attention and concentration performance [17]. Task IV investigated reaction time, mobility, speed and physical exercise by tapping touch sensors directed by signs on a screen (tapping test by talent-systems sportconsulting Gmbh, www.werthner.at) (Table 2a). All subjects performed the tasks after randomisation (www.randomizer.at) of the starting exercise. For example, subject one started with test I while subject two started with test II at the same time. After ten minutes working time and a five minute break, subject one carried on with task II and subject two with task I. After a five minute break, subject one started with task III while subject two performed task IV over a 10 minute period. Again after five minutes break, subject one continued with task IV and subject two with task III (Table 2b). Subjects documented their individual comfort and general condition in a structured assessment sheet before the tasks started and after every series of tasks (Table 2a). All four tasks were repeated 6 times at 22°C (6 hours total working time) on the first study day and four times at 28°C (4 hours total working time) on the second study day with waiting times between 4 and 21 days (mean waiting time 14 days). Limiting factors for working conditions were rated after every series from 1 to 10 on the assessment sheet whereby ranking 10 was a reason for terminating the study. Heart rate (HR) was measured with a wireless heart rate monitor placed below the sternum directly on the skin (Garmin Forerunner 305) during the task series. HR data before and after the task series was used for statistical analysis. Body temperature was assessed by tympanic infrared temperature measurement. Body weight was measured without PPE and undergarment at the beginning and after the last test (measurement accuracy 0.1kg) for calculating dehydration.

**Table 2:** Detailed description of tasks and serialisation.

**a) Task**	**Task Description**	**Readout**	**Mode**	**Position**
**I**	- closing coloured and numbered tubes with corresponding coloured and numbered screwtops together. - arranging closed tubes in a box following a certain pattern.	Comparison of total amount of handled tubes and wrong combinations.	Simulated glovebox	sitting
**II**	- pipetting a defined volume from three coloured water reservoirs into a 96-well plate following a certain amount and pattern.	Comparison of total amount of filled wells and wrong or omitted wells.	Simulated glovebox	sitting
**III**	**“d2 test of attention”** - ticking off every “d”-item with 2 bars in a pattern of “d” and “p” with different numbers and adjustments of bars. 14 rows with 47 items each have to be checked within 20 seconds per row (658 items in total).	A standardised matrix reveals correctly, wrongly or omitted “d”items.	Handwritten	sitting
**IV**	**Power of reaction test “TDS (test your talent)”** Digital readouts on a screen indicating to beat four touch sensors with the hands located on the left and right side in front of and behind the subject and two additional touch sensors on the floor for the legs.	Recording of reaction time.	Computer	standing
	**Assessment of individual perception:** subjective temperature sweating dizziness sickness headache hunger thirst subjective concentration view respiration urinary urgency fine motor skills mobility back pain other problems general condition	Ranging from 1 (low interference) to 10 (high interference, resulting in termination of the study).	Handwritten	sitting
**b)**	**Task Schedule**			
**Time**		**Suit A**	**Suit B**	
10 min		Task I	Task II	
5 min		Break	Break	
10 min		Task II	Task I	
5 min		Break	Break	
10 min		Task III	Task IV	
5 min		Break	Break	
10 min		Task IV	Task III	
1 min		Break	Break	
3 min		Assessment	Assessment	
1 min		Break	Break	

### Primary outcome measures

All subjects wearing their randomly allocated suits participated in task series at 22° and 28°C.

Physical measurements: Heart rate, fluid loss and body temperature.

Measurement of error rates in task I.

Measurement of concentration (d2 Test, task III).

Measurement of reaction time (task IV).

Measurement of individual perception and wellbeing before and after all task series (assessment sheet).

### Data protection and privacy

Individual-related information connected with data generated was exclusively stored in a coded way in a database with restricted access and password protection. For contacting the subjects a separate contact database with restricted access and password protection was established. The data generated were exclusively used in a coded way for analysis and publication.

### Statistical analysis

Descriptive Statistics for all physical measurements, task performance and assessment data are given as mean and standard deviation (SD).

Physical Measurements: heart rate, body temperature measured by tympanic infrared thermometer, and fluid loss determined on the basis of body weight reduction were documented before and after the task series. Heart rate was additionally recorded during the whole study phase to identify the most challenging task and to allow comparison of different tasks, error rates and reaction times. For each of the two working temperatures the differences between the two suits regarding heart rate, body temperature and fluid loss were assessed separately with an analysis of covariance (ANCOVA). Prior to analysis it was checked if the assumptions for performing an ANCOVA were fulfilled.

Task performance data were recorded 6 times at 22°C, and 4 times at 28°C. Assessment data were recorded a total of 7 times at 22°C and 5 times at 28°C including once before the series started to provide a baseline result. For each of the two working temperatures we calculated a repeated measurements analysis of variances (rmANOVA) to assess the effects of the working time as a within subject factor, and suit (A, B) as a between subject factor on the amount of processed tubes and the amount of wrongly screwed or arranged tubes in task I. For exploratory purposes the same analysis was performed for the assessment data.

Task II could not be evaluated due to a technical failure in the plate reading system and was therefore excluded from statistical analyses. For the data of task III we calculated descriptive statistics. For task IV a repeated measurements analysis of variances (rmANOVA) to assess the effects between the series as a within subject factor, and suit (A, B) as a between factor on the measured reaction time was calculated as well as descriptive analysis for the graphic chart.

For data management, descriptive analysis and figures we used Microsoft Excel 2003 (Version 11 for Windows 2003. Redmond, Washington (US): Microsoft Corporation). Analyses of variances were performed with IBM SPSS Statistics (Release 20.0.0.2 2011. Armonk (NY), USA: International Business Machines Corporation). Within subject effects were corrected with Greenhouse-Geisser correction if indicated. P-values less than 0.05 were considered as statistically significant.

## Results

Nineteen volunteers, 10 male, 9 female, aged between 21 and 38 years, participated in this study. Three subjects (two with suit B, one with suit A) who terminated the study before the end of the task series were included in the analyses of tasks in which they have participated. Two of them terminated due to indication of score 10 on the assessment sheet after series 4 (paranasal sinus obstruction) and after series 5 (hunger) on the first study day at 22°C. These subjects continued the study on study day two at 28°C. One subject assigned to suit B resigned from the study after the task series at 22°C not because of physical stress but without giving reason.

### Physical parameters

Fluid loss (Fig 2c) was not statistically significant between suit A (-0.53 kg, SD 0.67) and suit B (-0.32 kg, SD 0.34) at 22°C (ANCOVA, p = 0.985). However, there was a higher but statistically not significant dehydration with suit A at 28°C (-1.27 kg, SD 0.62) compared to suit B (-0.59 kg, SD 0.34) (ANCOVA, p = 0.069).

ANCOVA showed no significant effects for suit (A, B) and working temperature (22°C, 28°C) on body temperature (Fig 2d), although a slight but not significant decrease in body temperature for suit B at working temperature 22°C (-0.51°C, SD 0.85) was observed.

Heart rate (Fig 2e) showed a slight decrease for both suits at 22°C for the whole duration of all task series. At 28°C there was an average increase of heart rate of 15.3 (SD 19.81) beats per minute wearing suit A and 10.4 (SD 14.15) beats per minute wearing suit B (ANCOVA, p = 0.724).

### Task performances

#### Task I

**Fig 3:**
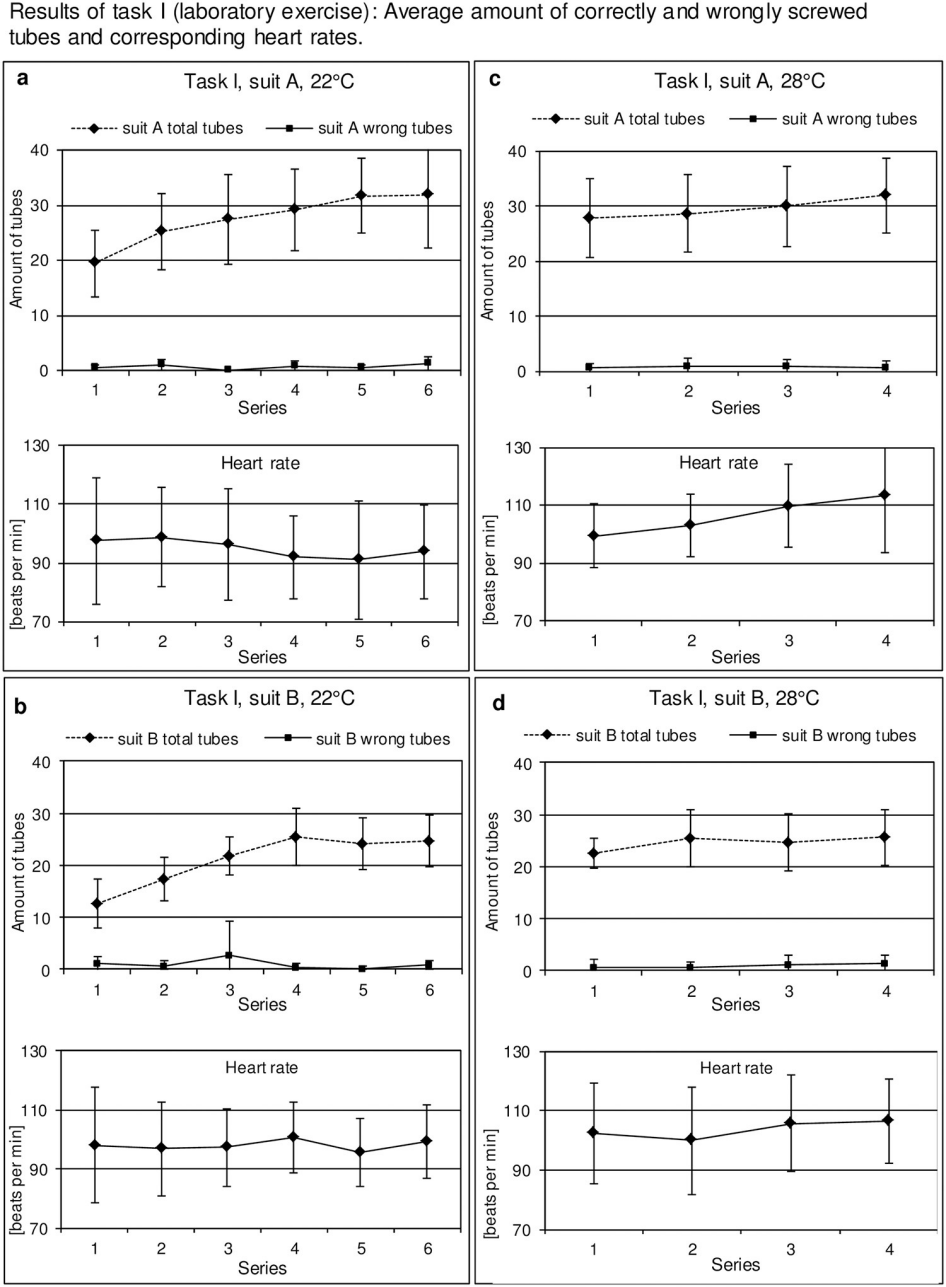
Results of task I (screwing tubes). Graphs show mean values and standard deviations calculated from the amount of all processed and wrongly screwed tubes (Y-axis) for 6 series (X-axis) by the subjects under the indicated conditions. (a) Suit A at 22°C, 6 series. (b) Suit B at 22°C, 6 series. (c) Suit A at 28°C, 4 series. (d) Suit B, 28°C, 4 series. The corresponding heart rates during the various task series are shown in the corresponding panels below.

At each working temperature the amount of processed tubes increased significantly from series to series in both suits (rmANOVA, p < 0.001 for 22°C and p = 0.013 for 28°C) (Fig 3). The amount of processed tubes was significantly higher when subjects were wearing suit A compared to suit B at 22°C (rmANOVA, p = 0.030), and a similar tendency was observed at 28°C (rmANOVA, p = 0.094). The amount of wrong tubes (wrong position or wrong cap) was constantly low for both suits at both temperatures. The heart rate was stable during the whole performance for suit B at both temperatures and for suit A at 22°C, whereas an increase was measured for suit A in individuals performing at 28°C.

#### Task III

**Fig 4:**
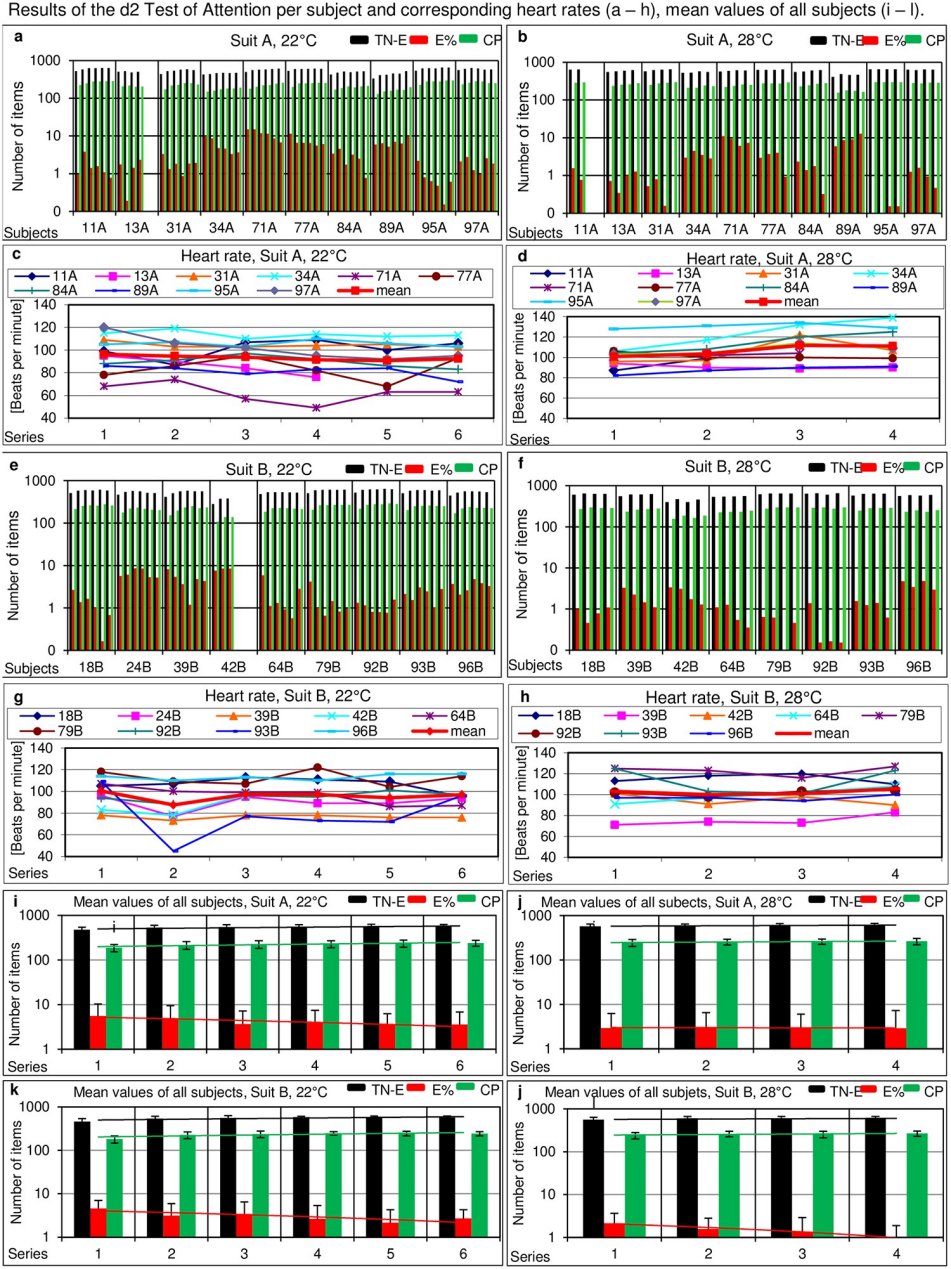
Results of the d2 test of attention. a, b, e, f: Y-axis: Graphs show TN-E (black bars), the amount of symbols recognized correctly, E% (red bars), the percentage of errors and CP (green bars), index of concentration performance. X-axis: Individual performance values of each subject performing six series at 22°C with (a) suit A, (e) suit B and four tests at 28°C (b) suit A and (f) suit B are divided by vertical lines. c, d, g, h: Courses of every individual´s heart rate corresponding to d2 test performances during the six or four tests, respectively. i, j, k and l: Mean values of all subjects calculated per test series including a trend line showing the average development of TN-E and E% in 6 series at 22°C and 4 series at 28°C.

Working rate, correctness and accuracy of discrimination were measured and evaluated by the d2 Test of Attention. The following characteristic numbers were calculated: 658 items per series was the maximum number. TN is the total number of items processed. E (error score) is the sum of all mistakes including E1 (errors of omission) and E2 (errors of commission, i.e. wrongly identified character, wrong dashes, wrong letters). E% is calculated as the proportion of errors made (E) within the number of all items processed. TN-E is the total number of items processed minus error score E. CP is the index of concentration performance calculated as the difference of correct items and errors of commission (E2) (12, 13). This number was at least constant or even increased with duration of the working time (Fig 4). TN-E was almost constant for every participant in all series. An increase of TN-E was observed from the first to the second or third series for almost all participants at 22°C working temperature. This tendency was not observed at 28°C. E% decreased over the first four series and remained constant until the 6^th^ series for 5 subjects at 22°C. Four subjects showed an increase of E% in the last two series. At 28°C the trend of decreasing E% was not observed for subjects wearing suit A. Heart rates at 22°C were below 100 beats per minute for both suits. At 28°C subjects with suit A showed an average increase of heart rate from 100 to 110 beats per minute. No increase was obvious when subjects wore suit B. Mean values of heart rates were calculated for each subject at each time point (Fig 4c, 4d, 4g and 4h). Mean values of TN-E, E% and CP of all subjects at each time point were calculated and connected with a trend line (Fig 4i, 4j, 4k and 4l). Trend line of CP shows a slight increase from the first to the last series, and E% a clear decrease.

#### Task IV

**Fig 5:**
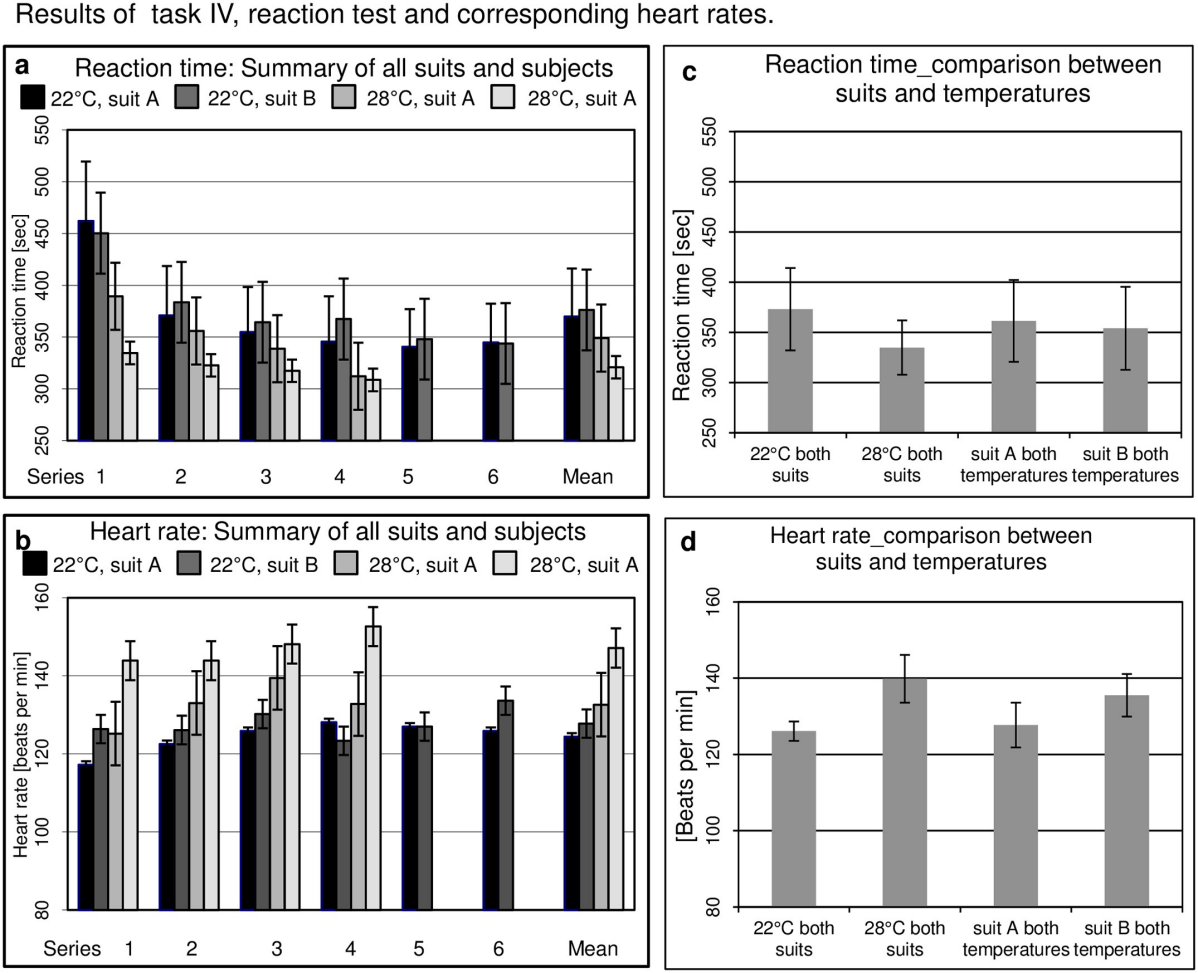
Task IV. Reaction time. Reaction time was evaluated by digital readouts on a screen indicating to tap four touch sensors with the hands located on the left and right side in front of and behind the subject and two touch sensors for the feet.

A general reduction in reaction time between the first and the following series was observed for both suits at 22°C (rmANOVA, p < 0.001) (Fig 5a) indicating a major training effect. There was also an improvement in the series at 28°C which was significantly more pronounced with suit A than suit B (rmANOVA interaction, p = 0.016). The heart rate of subjects wearing suit B at 28°C was significantly higher than those in suit A (Fig 5b). Mean values showed a shorter reaction time (Fig 5c) and a higher heart rate (Fig 5d) at 28°C compared to 22°C.

#### Assessment of individual perception of comfort and general condition

**Fig 6:**
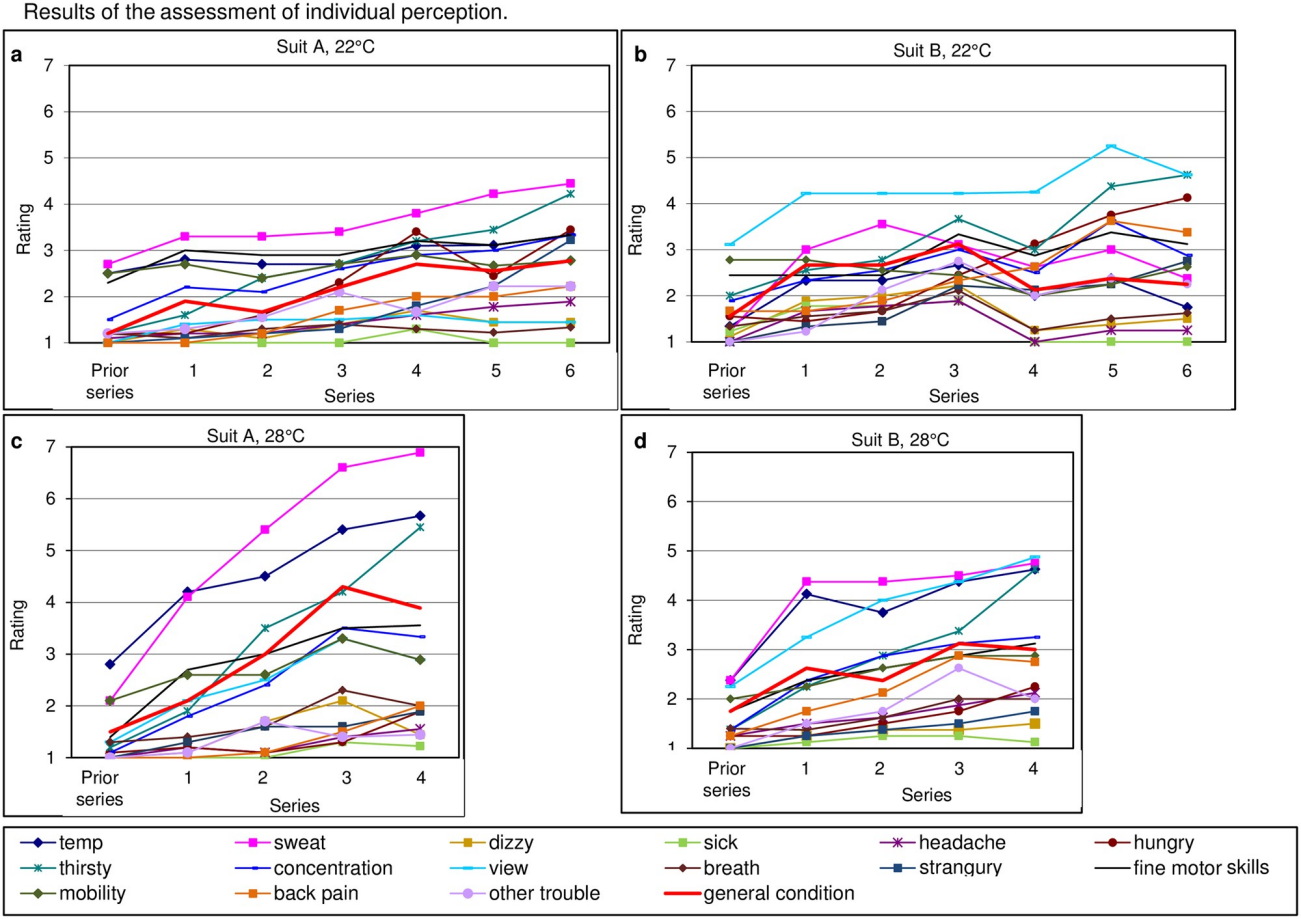
Results of the assessment of individual perception. Participants rated their individual comfort and general condition in an assessment sheet prior to and after the task series from 1 (= low interference) to 10 (= high interference, cause for termination).

The assessment sheets comprised fifteen different statements of individual perception of comfort and general condition documented, in a range from 1 (low interference) to 10 (high interference leading to termination of the study). Assessment sheets were completed before and after each task series at 22°C and 28°C, respectively (Fig 6). At 22°C we found no major effects for the parameters dizziness, thirst, concentration, restricted respiration, need for toilet break (urinary urgency), fine motor skills, mobility or other problems. Considerable but not significant differences were seen for temperature perception, sweating, headache and hunger, which were more pronounced for suit A. The view was considered more restricted by the flexible face shield of suit B than the fixed shield of suit A (rmANOVA, p < 0.001) at 22°C and this difference became more pronounced with proceeding working time (rmANOVA interaction, p < 0.021). Back pain was noted to be more pronounced with suit B than suit A, but this parameter did not show statistical significance (p = 0.096).

At 28°C working temperature, the view was again rated to be more restricted with suit B compared to suit A (rmANOVA, p = 0.027). Discomfort due to sweat increased in both suits (p < 0.001) and significantly more with suit A than with suit B (rmANOVA interaction, p = 0.003) but was still tolerable.

## Discussion

Based on the facts that wearing full body suits and powered-air-purifying-respirators (PAPR) protect from exposure to pathogens but constrict mobility, view and cause heat stress we tested whether these restraints could impact, for example, on concentration and increase in error rates due to fatigue. The combined evaluation of biophysical conditions and working performance of test persons should provide data on usability of PPE which can be easily and widely implemented in health care for bio-hazard protection [3].

Measurable physical parameters, for example, fluid loss were dependent on working temperature. At 28°C the average loss of fluid was higher, although not statistically significant. (Fig 2c). An unexpected observation was the slight (statistically not significant) decrease of body temperature when subjects were wearing the total body ventilated suit B at 22°C but not for the head ventilated suit A. Although tympanic infrared body temperature measurement may show some variability this decrease might indicate a cooling effect due to the continuous ventilation of the whole suit for 6 hours at 22°C. This difference was not observed at 28°C where the mean body temperature was the same for both suits (Fig 2d). An increase of body temperature over 39°C, which is a sign of dangerous heat stress [18] was not observed at any of the conditions tested. The decrease of heart rates at 22°C, which however did not show statistical significance, was not an expected physiological outcome. This could indicate that there is a certain stress level when donning the equipment for the first time leading to increased heart rates at the beginning of the study, which decreased again after adaptation to the conditions. The increased heart rates at 28°C with both suits indicate increased heat stress which was expected (Fig 2e). However, the increase in body temperature as well as the fluid loss was perceived according to the individual perception as moderate to high stress which was not a limiting factor even after 4 hours of working at the high temperature of 28°C.

Analysis of task I for both suits and both working temperatures revealed an increase of correct tubes and consequently a better ratio of total to wrong tubes with increasing numbers of the task series up to 6 and 4 hours of working, respectively. This indicates that effects of fatigue had less impact than the training effect under these experimental conditions. Since the training effect was also seen at the second series of tasks performed on another day at the higher temperature of 28°C we conclude that training effects did not interfere with general results of the tasks performed but rather serve as a relative indicator for stress and fatigue intensity. There was, however, a tendency towards differences between suit A and B with respect to the total number of correctly processed tubes which was higher for suit A. This difference is most likely explained by improved dexterity due to better fitting gloves for suit A than B as shown in the assessment of fine motor skills (Fig 6, fine motor skills).

Similar observations to those in task I were made for the d2 test (task III). Individual performances (Fig 4a, 4b, 4e and 4f) demonstrated that some subjects decreased their errors (red bars) and raised their index of concentration (green bars) with increasing numbers of task series (Fig 4i, 4j, 4k and 4l). Significant differences in the performance of the d2 test were observed neither for the two suit types nor for the two working temperatures.

Results of the reaction test (task IV) also demonstrated that fatigue effects were less pronounced than training effects, showing continuous decrease of reaction times with increasing number of task series. As this test required physical activity, low reaction time indicated ambitious activity and corresponded with high heart rates; and both parameters were further mirrored by higher temperature perception. The heart rate of subjects wearing suit B at 28°C was higher than for suit A (Fig 5b). This could be attributed to either increased physical activity as indicated by the shorter reaction time or increased work load of exercising in a fully ventilated suit (suit B). Surprisingly, mean values of reaction times (Fig 5c) for both suits showed lower reaction times at 28°C than 22°C.

Assessment of restrictions and discomfort due to the different suit types revealed that for suit B, restricted view caused by the flexible face shield was the most relevant parameter. Furthermore the respiration system worn as a rucksack led to back pain. In contrast, study participants reported for suit A increased temperature and sweating as the most pronounced factors which was expected for this suit with head ventilation. None of these parameters reduced the performance nor did they result in earlier termination of the study.

We did not evaluate the maximum possible working time since six hours at 22°C and four hours at 28°C were longer than the maximum allowed working periods (typically maximal four hours at 22°C) in positive pressure suited BSL4 laboratories. As a limitation it must be acknowledged that 9 out of 10 subjects allocated to suit A and 5 out of 9 subjects allocated to suit B indicated previous experience with PPE in general, more precisely, the use of any kind of protective suits but without PAPR.

In our study the use of PAPR providing constant ventilation and cooling of the head might have increased the tolerability of heat stress which probably would not have been the case with face masks or unpowered respirators [19]. The comparison of PAPR with other devices was not the focus of this study. A preferred use of PAPR is further underlined by increased safety due to reduced risk of unintentional contamination of the face. PAPR are also recommended by WHO, the US Centers of Disease Control and Prevention, and the Public Health Agency of Canada to protect from airborne infections (e.g. tuberculosis) Ebola virus disease and toxic aerosol generating procedures [20]. There are however reports with in part contradictory conclusions concerning possible restrictions caused by PAPR. Studies from the 1990s [21, 22, 23] stated no change in cognitive performance while wearing respirators, contradicting a study from 2013 [24] that ascertained a significant effect on errors made during cognitive tasks by wearing a full-face respirator.

## Conclusions

In conclusion, both suit types were well tolerated when performing different tasks related to sample processing and analyses necessary when protection of HCWs by PPE is required. The combined evaluation of physical parameters and subjective perception of restrictions and discomfort in task series was informative for identifying limiting factors for working in different types of PPE and should generate trust and confidence of personnel for working in PPE. Furthermore, data generated on the impact of wearing PPE under prolonged and stressful working conditions on error rates should be considered in defining working procedures and safety measures. This study can add to data about the impact of PPE on health care worker performance and comfort which may be of value for future pandemics.

## Supporting information

S1 File Study protocol original German. (PDF)

S2 File Study protocol English. (PDF)

S3 File Study protocol amendment original German. (PDF)

S4 File Study protocol amendment English. (PDF)

S5 File CONSORT checklist. (PDF)

S1 Table Subject´s raw data. This table provides general information about the subjects, all measured performance data of tasks I to IV and the data recorded from the assessment sheets.
(XLSX)

## References

[1] Castle N, Owen R, Hann M, Clark S, Reeves D, Gurney I. Impact of chemical, biological, radiation, and nuclear personal protective equipment on the performance of low- and high-dexterity airway and vascular access skills. Resuscitation. 2009;80(11): 1290–5. 10.1016/j.resuscitation.2009.08.001 19709794

[2] Kümin D, Krebs C, Wick P. How to Choose a Suit for a BSL-4 Laboratory- The Approach Taken at SPIEZ LABORATORY. Applied Biosafety. 2011;16 (2): 94–102.

[3] O´Brian C, Blanchard L, Cadarette B, Endrisick T, Xu X, Berglund L. Methods of Evaluating Protective Clothing Relative to Heat and Cold Stress: Thermal Manikin, Biomedical Modeling and Human Testing. J Occup Environ Hyg. 2011;8: 588–599. 10.1080/15459624.2011.613291 21936698

[4] Merens A, Cavallo JD, Thibault F, Salicis F, Munoz JF, Courcol R, et al Assessment of the bio-preparedness and of the training of the French hospital laboratories in the event of biological threat. Euro Surveill 2012;17(45):20312 23153476

[5] Garner A, Laurence H, Lee A. Practicality of performing medical procedures in chemical protective ensembles. Emerg Med Australas 2004;16(2): 108–13. 10.1111/j.1742-6723.2004.00560.x 15239724

[6] MacDonald RD, LeBlanc V, McArthur B, Dubrowski A. Performance of resuscitation skills by paramedic personnel in chemical protective suits. Prehosp Emerg Care 2006;10(2): 254–9. 10.1080/10903120500541076 16531385

[7] Steward JA, Lever MS. Evaluation of the operator protection factors offered by positive pressure air suits against airborne microbiological challenge. Viruses. 2012;4(8): 1202–11. 10.3390/v4081202 23012620 PMC3446757

[8] Zamora JE, Murdoch J, Simchison B, Day AG. Contamination: a comparison of 2 personal protective systems. Can Med Assoc J 2006;175(3): 249–54. 16880444 10.1503/cmaj.060094 PMC1513425

[9] Li L, Gu J, Shi X, Gong E, Li X, Shao H, et al Biosafety level 3 laboratory for autopsies of patients with severe acute respiratory syndrome: principles, practices, and prospects. Clinical Inf Dis 2005;41(6): 815–21. 10.1086/432720 PMC7107885 16107979

[10] Mertens M, Schmidt K, Ozkul A, Groschup MH. The impact of Crimean-Congo hemorrhagic fever virus on public health. Antiviral Res. 2013;98(2): 248–60. 10.1016/j.antiviral.2013.02.007 23458713

[11] Wölfel R, Stoecker K, Fleischmann E, Gramsamer B, Wagner M, Molkenthin P, et al Mobile diagnostics in outbreak response, not only for Ebola: a blueprint for a modular and robust field laboratory. Euro Surveill. 2015;20(44). 10.2807/1560-7917.ES.2015.20.44.30055 26559006

[12] Honda H, Iwata K. Personal protective equipment and improving compliance among healthcare workers in high-risk settings. Curr Opin Infect Dis 2016, 29: 400–406. 10.1097/QCO.0000000000000280 27257793

[13] CDC. Guidance on Personal Protective Equipment (PPE) To Be Used By Healthcare Workers during Management of Patients with Confirmed Ebola or Persons under Investigation (PUIs) for Ebola who are Clinically Unstable or Have Bleeding, Vomiting, or Diarrhea in U.S. Hospitals, Including Procedures for Donning and Doffing PPE. 2015. https://www.cdc.gov/vhf/ebola/healthcare-us/ppe/guidance.html.

[14] Hersi M, Stevens A, Quach P, Hamel C, Thavorn K, Garritty C, et al Effectiveness of Personal Protective Equipment for Healthcare Workers Caring for Patients with Filovirus Disease: A Rapid Review. PLoS One. 2015 10 9;10(10):e0140290 10.1371/journal.pone.0140290 eCollection 2015. 26451847 PMC4599797

[15] Sprecher AG, Caluwaerts A, Draper M, Feldmann H, Frey CP, Funk RH, et al Personal Protective Equipment for Filovirus Epidemics: A Call for Better Evidence. J Infect Dis. 2015:212 (Suppl 2). 10.1093/infdis/jiv153 PMC4564541 25821225

[16] Brickenkamp R ZE. d2 Test of Attention. Huber Ha, editor. Göttingen,Germany 1998.

[17] Zillmer EA, Kennedy CH. Construct validity for the d2 Test of Attention. Arch Clin Neuropsychol. 1999;8: 728–735.

[18] CDC. Interim Guidance for Healthcare Workers Providing Care in West African Countries Affected by the Ebola Outbreak: Limiting Heat Burden While Wearing Personal Protective Equipment (PPE). 2015. https://www.cdc.gov/vhf/ebola/hcp/limiting-heat-burden.html.

[19] Liverman CT, Domnitz SB, McCoy MA. "Front Matter." The Use and Effectiveness of Powered Air Purifying Respirators in Health Care: orkshop Summary. Washington, DC: The National Academies Press 2015. 25996018

[20] MacIntyre CR, Chughtai AA, Seale H, Richards GA, Davidson PM. Respiratory protection for healthcare workers treating Ebola virus disease (EVD): are facemasks sufficient to meet occupational health and safety obligations? Int J Nurs Stud. 2014;51(11): 1421–1426. 10.1016/j.ijnurstu.2014.09.002 25218265 PMC7126049

[21] Caretti DM. Cognitive Performance during Long-Term Respirator Wear While at Rest. Am Ind Hyg Assoc J. 1997;58(2): 105–109. 10.1080/15428119791012928 9042703

[22] Caretti DM. Cognitive Performance and Mood during Long-Term Respirator Wear and Exercise. Am Ind Hyg Assoc J. 1999;60(2): 213–218. 10.1080/00028899908984438 10222572

[23] Zimmermann NJ, Eberts C, Salvendy G., McCabe G. Effects of Respirators on Performance of Physical, Psychomotor and Cognitive Tasks. Ergonomics. 1991;34(3): 321–334. 10.1080/00140139108967316 2055218

[24] AlGhamri AA, Murray SL, Samaranayake VA. The effects of wearing respirators on human fine motor, visual, and cognitive performance. Ergonomics. 2013;56(5): 791–802. 10.1080/00140139.2013.767383 23514088

