# Supplementary material for: Limiting factors for wearing personal protective equipment (PPE) in a health care environment evaluated in a randomised study
Source: PLoS One. 2019 Jan 22;14(1):e0210775. doi: 10.1371/journal.pone.0210775 (PMC6342303; doi:10.1371/journal.pone.0210775)
Supplement: S1 File — (PDF) [file pone.0210775.s001.pdf]

STUDIENPROTOKOLL

TESTUNG VON PERSÖNLICHER SCHUTZAUSRÜSTUNG (PSA) FÜR EIN BSL4 UMFELD

Univ. Prof. Dr. Kurt Zaloukal
Mag. Martina Loibner
Mag. (FH) Sandra Hagauer

Institut für Pathologie
A-8036 Graz, Auenbruggerplatz 25

Graz, 04.04.2011

Zusammenfassung

Der Zweck dieser Studie ist es herauszufinden, wie sich das Tragen von Persönlicher Schutzausrüstung über einen längeren Zeitraum bei unterschiedlichen Temperaturbedingungen auf körperliche Leistungsfähigkeit, subjektives Befinden, Konzentrationsfähigkeit und Fehlerraten auswirkt. Zwei verschiedene Anzugsysteme werden während typischer Labortätigkeiten, bei zwei verschiedenen Temperaturbedingungen, miteinander verglichen. Um optimale Arbeitsbedingungen für zukünftige Mitarbeiter eines Hochsicherheitslabors zu entwickeln, ist es notwendig die genannten Parameter, sowie Indikatoren, die rechtzeitig auf Überbelastung und Fehlerraten hinweisen, zu identifizieren. Aus diesen Messungen soll auch eine maximal zumutbare Arbeitszeit unter den gegebenen Bedingungen hervorgehen. Durchgeführt wird diese Studie im Rahmen des EU-Projektes „European Research Infrastructure on Highly Pathogenic Agents (ERINHA)“, in dem eine gesamteuropäische Lösung für die Sammlung pathogener Proben entwickelt werden soll.

Administrative Struktur

Funktion	Name	Beruf
Studienleitung<br>Analytik	Univ. Prof. Dr. Kurt Zatloukal	Pathologe
Studienkoordination<br>Datenmanagement	Mag. (FH) Sandra Hagauer	Gesundheitsmanagerin<br>Dissertantin
Probenmanagement<br>Analytik	Mag. Martina Loibner	Mikrobiologin<br>Dissertantin

Einleitung

Im Rahmen des Campus Projektes der Medizinischen Universität Graz ist ein gemeinsames Labor für biologische Arbeiten im Risikobereich 3 für die Institute Pathologie, Hygiene, Mikrobiologie & Umweltmedizin und die Biobank vorgesehen. Im Kontext von Gesundheitsversorgung und medizinischer Diagnose soll diese Einheit für die sichere und rasche Durchführung von Autopsien und Probensammlungen zur Erforschung von Pathogen-Host-Interaktionen zur Verfügung stehen. Um auch Erkrankungen durch Erreger mit pandemischem Potential bzw. therapieresistente Errerger, deren Risikogrupppe noch nicht bekannt ist, bearbeiten zu können, soll dieses Labor zusätzlich mit mobilen Isolationseinheiten (Gloveboxen), sowie einem erhöhten Personenschutz ausgestattet werden. Diese Art der Probensammlung ist derzeit nicht organisiert, jedoch von großer Bedeutung um bei neuauftretenden Erkrankungen durch Pathogenen festellen zu können, ob die Mortalität auf den Erreger oder auf Komorbiditäten zurückzuführen ist. Zusätzlich können dadurch wichtige Informationen, die sich auf die Organspezifität von Erregern, Pathogenitätsmechanismen, sowie Abwehrreaktionen des Körpers beziehen, gewonnen werden.

Der Aufbau eines derartigen Probensammelzentrums erfolgt im Rahmen einer europaweiten Infrastrukturinitiative der europäischen Kommission (ERINHA = European Research Infrastructure on Highly Pathogenic Agents). Eine der Aufgaben im Zusammenhang mit diesem Projekt ist es, ein Gesamtkonzept für eine universell einsetzbare Probensammlung, im Rahmen von Autopsien, zu etablieren. Dieses Konzept soll an der Schnittstelle zwischen Gesundheitssystem und Hochsicherheitslabor angesiedelt sein und als Modelllösung für Gesamteuropa geeignet sein. Aus diesem Grund ist es erforderlich dass dieses Konzept auch für den mobilen Einsatz insbesondere für Länder mit heißem Klima einsetzbar ist (z.B. Südosteuropa, Afrika). Die Planung findet auch in enger Zusammenarbeit mit dem HITT (High Infectious Transport Team) des Roten Kreuzes, sowie der Landessanitätsdirektion und der Abteilung für Katastrophenschutz des Landes Steiermark, statt.

Bei der Erstellung eines Probensammlungsmodells ist es neben dem Schutz von Mensch und Umwelt vor ungewollter Freisetzung von humanpathogenen Krankheitserregern wichtig, das Laborpersonal gegen Infektionen zu schützen. Um einen ausreichenden Schutz gewährleisten zu können, sind folgende Barrierestufen vorgesehen:

1. Räumliche Infrastruktur: P3-Labor
2. Raumausstattung: Gloveboxen mit Unterdruck und Schleusensystem, Personenschleuse mit Dekontaminationsmöglichkeit
3. Persönliche Schuztausrüstung: keimdichte Ganzkörperschutzanzüge, Beatmungssystem mit HEPA-Filter, Schutzhandschuhe, Schutzstiefel

Ziele der Studie

Diese Studie konzentriert sich vorwiegend auf die 3. Barrierestufe. Ziel dieser Studie ist es, herauszufinden wie sich das Tragen von Persönlicher Schutzausrüstung über einen längeren Zeitraum bei unterschiedlichen Temperaturbedingungen auf körperliche Leistungsfähigkeit, subjektives Befinden, Konzentration und Fehlerraten auswirkt (erhöhte Umgebungstemperatur könnte einen limitierenden Faktor beim Arbeiten mit erhöhtem Personenschutz darstellen). Weiters stellt sich die Frage welche maximal mögliche Arbeitszeit sich daraus ergibt. Es ist anzunehmen, dass mit zunehmender Beeinträchtigung die Fehlerraten ansteigen, Schutzvorschriften daher nicht mehr eingehalten werden können und in der Folge die Verletzungsgefahr somit massiv erhöht wird. Aus diesem Grund ist es wichtig, Indikatoren, die rechtzeitig auf einen Konzentrationsabfall hinweisen, zu identifizieren.

Studiendesign

Bei dieser Studie handelt es sich um eine monozentrisch durchgeführte, randomisierte, cross-over Studie. Im Rahmen der Studie werden zwei unterschiedliche Schutzanzugsysteme, bei jeweils zwei unterschiedlichen Temperaturbedingungen, gegenübergestellt. Diese Studie dient zur Ermittlung der körperlichen Leistungsfähigkeit, der Konzentrationsfähigkeit, von Fehlerraten und des subjektiven Befindens bei Laborarbeiten mit persönlicher Schutzausrüstung. Es ist eine Pilotphase geplant, die zur Optimierung der simulierten Laborarbeit in Gloveboxen, zur Definition möglichst aussagekräftiger Simulationsbedingungen (z.B. wieviele Pipettierschritte in einer bestimmten Zeit machbar sind, Testung der Logistik während der Durchführung), sowie zur Ermittlung der Relevanz der geplanten Messungen dienen soll. In die Pilotphase werden zwischen 5-7 freiwillige Probanden eingeschlossen. Basierend auf Erkenntnissen vorangegangener Studien, in Bezug auf Testung von persönlicher Schutzausrüstung, wird von einer Probandenzahl von ungefähr 20 Personen ausgegangen, wobei aufgrund des relativ hohen Zeitaufwandes für die Studie mit einer Dropout-Rate von ca. 50% gerechnet werden muss.

Geschätzter Zeitplan

**PILOTPHASE**	**BIS**
Probandenrekrutierung	Mitte Mai
Testlauf	Mitte Mai
Auswertung	Ende Mai
**DURCHFÜHRUNGSPHASE**	**BIS**
Probandenrekrutierung	Mitte Mai
Modul I a / b ~10 Proband/Innen	Anfang Juni
Zwischenauswertung	Mitte Juni
Modul I a / b ~10 Proband/Innen	Mitte Juni
Auswertung Modul I	Ende Juni
Modul II a / b ~10 Proband/Innen	Ende Juni
Zwischenauswertung	Mitte Juli
Modul II a / b ~10 Proband/Innen	Mitte Juli
Auswertung Modul II	Ende Juli
Endauswertung	Ende August

Studienpopulation

Die Studie unterteilt sich in eine Pilot- und eine Durchführungsphase, wobei in die Pilotphase zw. 5-7 und in die Durchführungsphase max. 20 freiwillige Proband/Innen eingeschlossen werden. Die Probandenrekrutierung für die Studie erfolgt über freiwillige Meldung aufgrund Auschreibung und Aushang an der KAGES, der Medizinische Universität, der Karl-Franzens-Universität, sowie der FH JOANNEUM und richtet sich vorwiegend an Student/innen und Mitarbeiter/innen dieser Institutionen. Die unten angeführten Ein- und Ausschlusskriterien kommen hier zur Anwendung.

Einschlusskriterien:

- Geschlecht: weiblich / männlich
- Alter: 18-65 Jahre
- gute psychische und physische Belastbarkeit

Ausschlusskriterien:

- Alter: <18 Jahre; >65 Jahre
- Schwangerschaft, Allergie gegen Latex bzw. PVC, Klaustrophobie, Hypotonie (Neigung zum Kollabieren), Infektionskrankheiten, Neigung zu Thrombosen
- nicht therapierte chronische Erkrankungen, wie z.B.: Asthma, COPD, sonstige Lungenerkrankung, Herz-Kreislauferkrankung, Epilepsie
- Teilnahme an einer anderen Studie

Methoden

Alle Studienteilnehmer testen je zwei Schutzanzugsysteme:

a) System A: Ganzkörperoverall mit Respirator
   - z.B. Tychem F Schutzanzug inkl. Socken
   - z.B. 3M™ Mehrwegleichthaube S-655
   - z.B. 3M™ Jupiter™ Gebläseeinheit
   - z.B. Sempermed OP-Handschuhe (optional)
   - z.B. Stiefeln

b) System B: Ganzkörperanzug mit ingegriertem Belüftungssyystem (Überdruck)
   - z.B. 3M™ JS-Serie Typ 3 Respiratory Protective Suit (CRPS)
   - z.B. Sempermed OP-Handschuhe (optional)
   - z.B. Stiefeln

bei unterschiedlicher Umgebungstemperatur (Modul I: ~ 20°C / Modul II: ~ 28°C) zu jeweils zwei verschiedenen Terminen. Zu jedem Termin führen zwischen zwei und vier Proband/Innen die unten angeführten Testreihen durch. Die Zuteilung mit welchem Schutzanzugsystem der/die Proband/in beginnt erfolgt über ein Randomisierungstool: http://www.randomizer.at/.

Die unten angeführten Testreihen laufen hintereinander ab, wobei die Proband/Innen zeitversetzt beginnen. Nach 15 Minuten wird zum nächsten Test gewechselt. Zwischen den einzelnen Testungen erfolgt eine 5-minütige Erholungspause. Die Tests 1-3 werden so lange wiederholt, so lange der/die Proband/In die gegebenen Bedingungen toleriert oder bis er aus anderen Gründen abbricht (Modul I: max. 6 Std. / Modul II: max. 4 Std.). Während der Testphasen werden Herzfrequenz (HF) / Herzfrequenzvariabilität (HFV), Sauerstoffsättigung (SpO_2) und Körperkerntemperatur gemessen und in regelmäßigen Abständen aufgezeichnet. Unmittelbar vor und nach den einzelnen Modulen werden Urin- und Speichelproben, zur Erstellung eines Metabolitenprofils entnommen, sowie Körpergewicht und Gewicht des Anzugsystems, zur späteren Berechnung des Flüssigkeitsverlustes, gemessen. Zusätzlich zu den genannten Messungen erfolgt eine ausführliche Einschulung, Aufklärung und Anamnese vor Beginn der Studie. Die Proband/Innen werden aufgefordert sich über ihr subjektives Befinden während der Durchführung der Testreihen zu äußern, zudem erfolgt eine stündliche Befragung mittels strukturiertem Fragebogen (es erfolgt eine durchgehende Aufzeichnung über das Kommunikationssystem).

MODUL I a/b (Umgebungstemperatur ~ 20°C)

Test	Tätigkeit	Art	Position
1	Laborübung I<br>- Probenröhrchen zusammenschrauben<br>- nach vorgegebenem Muster einordnen	simulierte Glovebox	sitzend
2	Laborübung II<br>- Flüssigkeit (z.B. Wasser) pipettieren<br>- nach vorgegebenen Mengen	simulierte Glovebox	stehend
3	Konzentrationstest	am PC od. schriftlich	sitzend

MODUL II a/b (Umgebungstemperatur ~ 28°C)

Test	Tätigkeit	Art	Position
1	Laborübung I<br>- Probenröhrchen zusammenschrauben<br>- nach vorgegebenem Muster einordnen	simulierte Glovebox	sitzend
2	Laborübung II<br>- Flüssigkeit (z.B. Wasser) pipettieren<br>- nach vorgegebenen Mengen	simulierte Glovebox	stehend
3	Konzentrationstest	am PC od. schriftlich	sitzend

Parameter	Messgerät	Einheit	vor Modul	während Modul	nach Modul
**Körpergröße**	Maßband	cm	X		
**Körpergewicht**	Personenwaage	kg	X		X
**Body-Mass-Index**	Taschenrechner	BMI	X		
**Blutdruck (RR)**	Blutdruckmessgerät	mm/Hg	X		X
**Urin**	NMR* (extern)		X		X
**Speichel**	NMR* (extern)		X		X
**Herzfrequenz/ -variabilität (HF/HFV)**	Pulsgurt od. Pulsoximeter od. EKG-Monitor	Schläge pro Minute	X	X	X
**O2-Sättigung (SpO2)**	Pulsoximeter	%	X	X	X
**Körpertemperatur**	Thermometer (tba)	°C	X	X	X
**Flüssigkeitsverlust**	Waage	ml			X

*Nuclear Magnetic Resonance (Erstellung eines Metabolitenprofils)

Studienablauf (Beschreibung eines typischen Versuchsablaufs)

I PILOTPHASE

a) Probandenrekrutierung

Aussendung / Aushang der Ausschreibung: MUG, KAGES, KFU, FH JOANNEUM
Erstellung Kontaktdatenbank für Interessenten
Einladung zur Informationsveranstaltung
Informationsveranstaltung (Alternativtermin bei Bedarf)
- Vorstellen der Studie
- Durchführung der Aufklärungsgespräche
- Ausfüllen der Anamnesefragebögen von den einzelnen Interessenten
- Ausgabe der Einverständniserklärung

Auswertung der Anamnesefragebögen
Übernahme der unterschriebenen Einverständniserklärungen
Einschluss in die Pilotphase / Studie
Terminkoordination für die Pilotphase
Terminkoordination für die einzelnen Module

b) Durchführung Pilotphase

Testung der Anzüge
Testung der Laborübungen / des Konzentrationstests
Auswertung auf Basis subjektiver Beurteilung

c) Optimierung des Studienprotokolls lt. Ergebnissen d. Pilotphase

II DURCHFÜHRUNGSPHASE

a) Durchführung Modul I a/b + II a/b

Vorbereitung

Vergabe einer Probanden-ID
Übergabe des randomisiert gewählten Schutzanzugsystems
Kurze Einführung
-> nur beim 1. Termin (Anziehen des Anzuges, Testreihen erklären,…)

Eingangsmessungen (unmittelbar vor Beginn der Testreihen)

Anamnese (modulbezogen)
Sammlung Speichelprobe -> gekühlte Lagerung
Sammlung Urinprobe (komplette Blasenentleerung) -> gekühlte Lagerung
Messung HF (Eintrag in Datenbank)
Messung SpO_2 (Eintrag in Datenbank)
Messung Körpertemperatur (Eintrag in Datenbank)
Messung Blutdruck (Eintrag in Datenbank)
Messung Körpergewicht (ohne Anzug)
Messung Gewicht Anzug
Anbringen des HF-Messgerätes / Pulsoximeters
Anbringen des Temperaturmessgerätes
Anziehen des Schutzanzugs
Messung Gesamtgewicht (Proband/In inkl. Anzug)

Durchführung der Testreihen

Zyklus I
Laborübung I (Dauer: 15 Min. + Erholungspause: 5 Min.)
Laborübung II (Dauer: 15 Min. + Erholungspause: 5 Min.)
Aufzeichnung der Messdaten (HF, SpO_2, °C)
Konzentrationstest (Dauer: 15 Min. + Erholungspause: 5 Min.)
Zyklus II
Laborübung I (Dauer: 15 Min. + Erholungspause: 5 Min.)
Laborübung II (Dauer: 15 Min. + Erholungspause: 5 Min.)
Aufzeichnung der Messdaten (HF, SpO_2, °C)
Konzentrationstest (Dauer: 15 Min. + Erholungspause: 5 Min.)
Zyklus III
Laborübung I (Dauer: 15 Min. + Erholungspause: 5 Min.)
Laborübung II (Dauer: 15 Min. + Erholungspause: 5 Min.)
Aufzeichnung der Messdaten (HF, SpO_2, °C)
Konzentrationstest (Dauer: 15 Min. + Erholungspause: 5 Min.)
Zyklus IV
Laborübung I (Dauer: 15 Min. + Erholungspause: 5 Min.)
Laborübung II (Dauer: 15 Min. + Erholungspause: 5 Min.)
Aufzeichnung der Messdaten (HF, SpO_2, °C)
Konzentrationstest (Dauer: 15 Min. + Erholungspause: 5 Min.)
Zyklus V (nur Modul I)
Laborübung I (Dauer: 15 Min. + Erholungspause: 5 Min.)
Laborübung II (Dauer: 15 Min. + Erholungspause: 5 Min.)
Aufzeichnung der Messdaten (HF, SpO_2, °C)
Konzentrationstest (Dauer: 15 Min. + Erholungspause: 5 Min.)
Zyklus VI (nur Modul I)
Laborübung I (Dauer: 15 Min. + Erholungspause: 5 Min.)
Laborübung II (Dauer: 15 Min. + Erholungspause: 5 Min.)
Aufzeichnung der Messdaten (HF, SpO_2, °C)
Konzentrationstest (Dauer: 15 Min. + Erholungspause: 5 Min.)

Endmessungen (unmittelbar nach Beendigung od. Abbruch des Testreihen)
Messung Gesamtgewicht (Proband/In inkl. Anzug)
Ausziehen des Schutzanzugsystems
Messung Körpergewicht (ohne Anzug)
Messung Gewicht Anzug
Entfernung des HF-Messgeräates / Pulsoximeters
Entfernung des Temperaturmessgerätes
Sammlung Speichelprobe -> gekühlte Lagerung
Sammlung Urinprobe -> gekühlte Lagerung
Messung HF (Eintrag in Datenbank)
Messung SpO_2 (Eintrag in Datenbank)
Messung Körpertemperatur (Eintrag in Datenbank)
Messung Blutdruck (Eintrag in Datenbank)

b) Datenauswertung
Exportieren der codierten Probandendaten von Access in Excel
Weitergabe der codierten Daten an Statistiker
Interpretation der Daten
Erstellen der Publikation
Veröffentlichung der Publikation in einer Fachzeitschrift / Dissertation

Studienabbruch

Der/die Proband/in kann die Studie jederzeit abbrechen (Gründe müssen nicht, können jedoch angegeben werden). Im Modul I, in der die Testungungen bei einer Umgebungstemperatur von ca. 20°C stattfinden, wird nach Ablauf von max. 6 Stunden automatisch beendet. Im Modul II, in der die Testungen bei einer Umgebungstemperatur von ca. 28°C stattfinden, wird nach Ablauf von max. 4 Stunden automatisch beendet.

Therapiesicherheit

Für die in die Studie eingeschlossenen Proband/Innen entsteht weder ein Nutzen noch ein Risiko. Es könnte aufgrund der Dichtheit der verwendeten Schutzanzüge zu Belastungen wie Hitzestau und Flüssigkeitsverlust durch Wärmeentwicklung im Anzug kommen und somit unter Umständen zu Herz-Kreizlauf-Beschwerden (z.B. Kollabieren) führen. In diesem Fall wird umgehend ein in Rufbereitschaft stehender Arzt konsultiert. Ansonsten ist diese Studie mit keinen weiteren Risiken behaftet.

Datenmanagement

Das Datenmanagement der Probandendaten erfolgt ausschließlich in codierter Form über eine Accessdatenbank, welche nach einem Schema in dieser Art aufgebaut sein wird:

Anamnese
Probanden-ID
Geschlecht
Geburtsdatum
Anamnesedatum
Uhrzeit
Körpergröße
Körpergewicht
BMI
HF in Ruhe
SpO_2 in % in Ruhe
Körpertemperatur in °C in Ruhe
RR systolisch in Ruhe
RR diastolisch in Ruhe
Anmerkungen

Modul I a	Modul I b	Modul II a	Modul II b
Untersuchungsdatum	Untersuchungsdatum	Untersuchungsdatum	Untersuchungsdatum
Testbeginn	Testbeginn	Testbeginn	Testbeginn
Testende	Testende	Testende	Testende
Testzeit gesamt	Testzeit gesamt	Testzeit gesamt	Testzeit gesamt
Schutzanzug	Schutzanzug	Schutzanzug	Schutzanzug
Raumtemperatur	Raumtemperatur	Raumtemperatur	Raumtemperatur
Anmerkungen	Anmerkungen	Anmerkungen	Anmerkungen
**Regelmäßige Messungen während der Testungen**			
Uhrzeit	Uhrzeit	Uhrzeit	Uhrzeit
HF	HF	HF	HF
SpO_2	SpO_2	SpO_2	SpO_2
Körpertemperatur ° C	Körpertemperatur ° C	Körpertemperatur ° C	Körpertemperatur ° C
**Anfangs-/Endmessungen**			
RR systolisch	RR systolisch	RR systolisch	RR systolisch
RR diastolisch	RR diastolisch	RR diastolisch	RR diastolisch
Körpergewicht in g	Körpergewicht in g	Körpergewicht in g	Körpergewicht in g
Gewicht Anzug in g	Gewicht Anzug in g	Gewicht Anzug in g	Gewicht Anzug in g
Gesamtgewicht in g	Gesamtgewicht in g	Gesamtgewicht in g	Gesamtgewicht in g
Flüssigkeitsverlust in g	Flüssigkeitsverlust in g	Flüssigkeitsverlust in g	Flüssigkeitsverlust in g

Damit eine regelmäßige Kontaktaufnahme (für die unterschiedlichen Testmodule) mit den Proband/Innen möglich ist, werden die Kontaktdaten gemeinsam mit der Probanden-ID in einer separaten, passwortgeschützten Datenbank erfasst.

Kontaktdaten
Probanden-ID
Titel
Vorname
Nachname
Email
Telefon
Anmerkungen

Ethische Aspekte

Für die in die Studie eingeschlossenen Proband/Innen entsteht weder ein Nutzen noch ein Risiko. Es könnte aufgrund der Dichtheit der verwendeten Schutzanzüge zu Belastungen wie Hitzestau und Flüssigkeitsverlust durch Wärmeentwicklung im Anzug kommen und somit unter Umständen zu Herz-Kreislauf-Beschwerden (z.B. Kollabieren) führen. In diesem Fall wird umgehend ein in Rufbereitschaft stehender Arzt konsultiert. Ansonsten ist diese Studie mit keinen weiteren Risiken behaftet. Zur Abklärung der ethischen Aspekte wird ein Ethikkommissionsanstrag an die Ethikkommission der Medizinischen Universität Graz gestellt. Die Aufklärung der Proband/Innen erfolgt in Form einer (bei Bedarf wiederholten) Informationsveranstaltung in der auch die Einwilligungserklärung ausgegeben wird.

Datenschutz

Die personenbezogenen Angaben zu den in den Studien generierten Daten werden ausschließlich in codierter Form in einer Datenbank mit begrenzten Zugriffsrechten und eigenen Passwörtern gespeichert. Nur zur Kontaktaufnahme mit den Proband/Innen wird eigens eine Kontaktdatenbank, ebenfalls mit begrenzten Zugriffsrechten und eigenen Passwörtern erstellt. Die generierten Daten werden für Analyse, Publikationen bzw. für den Austausch mit Kooperationspartner ausschließlich in codierter Form verwendet. Weiters wird sichergestellt, dass Ergebnisse aus der Studie (wie z.B. das Abschneiden bei Tests) gegebenenfalls keinen Einfluss auf ein bestehendes Dienstverhältnis oder das Studium haben.

Fallzahlschätzung

Die Fallzahl von ungefähr 20 Proband/Innen wurde basierend auf Erkenntnissen vorangegangener Studien in Bezug auf Testung von Persönlicher Schutzausrüstung, geschätzt. Aufgrund des relativ hohen Zeitaufwandes für die Studie wird mit einer Dropout-Rate von ca. 50% gerechnet.

Berichterstattung

Die Ergebnisse sollen in wissenschaftlichen Journalen, in einer Dissertation, sowie auf Kongressen publiziert werden.

Literaturverzeichnis

Bundesamt für Bevölkerungsschutz (2007). *Biologische Gefahren I, Handbuch zum Bevölkerungsschutz,* Bonn: Druckpartner Moser Druck + Verlag GmbH, Rheinbach.

Bundesamt für Bevölkerungsschutz und Katastrophenhilfe (2007). *Biologische Gefahren II. Entscheidungshilfen zu medizinisch angemessenen Vorgehensweisen in einer B-Gefahrenlage,* Bonn: Druckpartner Moser Druck + Verlag GmbH, Rheinbach.

Bundesministerium für Arbeit, Soziales und Konsumentenschutz (2009). *Arbeitsstoffe: Biologische Arbeitsstoffe (Einstufung, Schutzmaßnahmen, Branchenbeispiele),* Wien.

Bundesministerium für Arbeit, Soziales und Konsumentenschutz (1998).*Verordnung der Bundesministerin für Arbeit, Gesundheit und Soziales über de Schutz der Arbeitnehmer/innen gegen Gefährdung durch biologische Arbeitsstoffe (Verordnung biologische Arbeitsstoffe - VbA),* Wien.

Fleming, D.O. & Hunt, D.L. (2006). *Biological Safety, Principles and Practices,* 4th Edition, Washington DC: ASM Press.

Li, L et al. (2005). Biosafety level 3 laboratory for autopsies of patients with severe acute respiratory syndrome: principles, practices, and prospects. Clinical infectious diseases : an official publication of the Infectious Diseases Society of America, 41(6), pp. 815-821.

Marklund, L.A. (2003). Patient care in a biological safety level-4 (BSL-4) environment. Critical care nursing clinics of North America, 15(2), pp. 245-255.

Anhang

Ethikkommissionsanstrag
Probandeninformation / Einwilligungserklärung
Nachweis der Qualifikation des Prüfers
Erklärung von Interessenskonflikten
Probandenausschreibung
Probandenvergütung

Unterschrift:……………………………….
(Prüfer)
