# Supplementary material for: Limiting factors for wearing personal protective equipment (PPE) in a health care environment evaluated in a randomised study
Source: PLoS One. 2019 Jan 22;14(1):e0210775. doi: 10.1371/journal.pone.0210775 (PMC6342303; doi:10.1371/journal.pone.0210775)
Supplement: S2 File — (PDF) [file pone.0210775.s002.pdf]

Study protocol

TESTING OF PERSONAL PROTECTIVE EQUIPMENT (PPE) FOR A BSL 4 ENVIRONMENT

Univ. Prof. Dr. Kurt Zatloukal
Mag. Martina Loibner
Mag. (FH) Sandra Hagauer

Institute of Pathology
A-8036 Graz, Auenbruggerplatz 25

Graz, 04.04.2011

Abstract

The aim of the study is to identify how the wearing of personal protective equipment (PPE) over a longer period of time under different temperature conditions affects physical performance, individual feeling, power of concentration and error ratio. Two different PPE systems are compared while carrying out typical laboratory work in two different temperature conditions. To be able to develop adequate working conditions for future laboratory workers in high security laboratories, it is necessary to identify previously mentioned parameters as well as indicators that duly indicate overload and error ratio. Based on these measurements a maximum reasonable working time under given circumstances shall be established. The study is conducted within the framework of the EU project „European Research Infrastructure on Highly Pathogenic Agents (ERINHA)“ where a pan-European solution for the collection of pathogenic samples should be developed.

Administrative structure

Function	Name	Profession
Principal Investigator<br>Analysis	Univ. Prof. Dr. Kurt Zatloukal	Pathologist
Study Coordination<br>Data Management	Mag. (FH) Sandra Hagauer	Health Management<br>PhD student
Sample<br>Management<br>Analysis	Mag. Martina Loibner	Microbiologist<br>PhD student

Introduction

Within the framework of the Campus project of the Medical University of Graz a joint laboratory for working with biological agents in risk group 3 is designated for the Institutes of Pathology, Hygiene, Microbiology and Environmental Medicine and Biobank. In the context of healthcare and medical diagnostics the mentioned unit should enable a safe and immediate execution of autopsies and sample collection to research pathogen-host-interactions. To be able to work on pathogens causing diseases with pandemic potential as well as therapy resistant pathogens whose risk group is unknown, the laboratory should additionally be equipped with a mobile isolation unit (glovebox) and should furthermore include increased personal protection. This kind of sample collection is currently not organised but is highly relevant to determine whether mortality can be traced back to the agent or to co-morbidities when diseases caused by pathogens are occurring. Furthermore important information concerning the organ specifity of pathogens, pathogenic mechanisms, as well as the body’s infection defense reactions can be gained.

The establishment of such a sample collection centre takes places within the framework of a pan-European infrastructure initiative of the European Commission (ERINHA = European Research Infrastructure on Highly Pathogenic Agents). One of the tasks linked to this project is the establishment of a master plan for an all-purpose sample collection during autopsy. This concept should be set up at the interface between the health system and high security laboratory and should be an appropriate mock-up solution for all of Europe. For this reason a mobile solution especially applicable in countries with a warmer climate (e.g. South-East-Europe, Africa) is required. The planning takes place in close collaboration with the High Infectious Disease Transport Team (HITT) of the Red Cross Styria, as well as the the Provincial Health Directorate Styria and the Department of Civil Protection Styria.

Beside safeguarding human beings and environment from unintentional disposal of pathogenic agents it is important to protect laboratory staff from infections. To ensure sufficient protection the following safety precautions are intended:

1. Spatial infrastructure: laboratory with security level 3
2. Room setup: gloveboxes with negative pressure and material lock, manlocks with opportunity for decontamination
3. Personal protective equipment: virus-tight-whole-body protective suits, respiratory devices with HEPA filters, virus-tight gloves and boots

Objective of the study

This study focuses predominantly on barrier level 3. Aim of the study is to identify how wearing PPE over a longer period of time in different temperature conditions affects physical performance, individual feeling, power of concentration and error ratio (elevated environmental temperature could pose a limiting factor while working with increased personal protection). Furthermore it raises the question of which possible maximum working timeis feasible. It is to assume that raising the time limit implicates higher error ratio, followed by the fact that protective provisions can’t be met, which furthermore highly increases risk of injury. For this reason it is important to identify indicators that point out loss of concentration in a timely manner.

Study design

This study is planned as a monocentric, randomised cross-over study. During the course of the study, two protective suits will be compared under two different temperature conditions. This study serves to determine the bodily performance, the concentration levels, error rates, and subjective well being during laboratory work in protective suits. A pilot phase is planned, in order to optimise the simulated laboratory work in glove boxes, to define the most meaningful simulated conditions (e.g., how many pipetting steps are possible in a given time, testing the logistics during the procedure), as well as to determine the relevance of the planned measurements. In the pilot phase, between 5-7 voluntary test subjects will be included. Based on the evidence from published studies regarding testing of personal protective equipment, a participant number of maximally 20 is assumed, although, due to the relatively long time required for the study, a dropout rate of about 50% is to be expected.

Estimated time schedule

**PILOT PHASE**	**End date**
Recruiting of subjects	mid May
Testing series	mid May
Evaluation	End of May
**EXECUTION PHASE**	**BIS**
Recruiting of subjects	mid May
Module I a / b ~10 subjects	Beginning of June
Interim analysis	mid June
Module I a / b ~10 subjects	id June
Evaluation module I	End of June
Module II a / b ~10 subjects	End of June
Interim evaluation	mid July
Module II a / b ~10 subjects	mid July
Evaluation module II	End of July
Final evaluation	End of August

Study population

The study is split into a pilot and execution phase, where 5-7 voluntary subjects are included in the pilot phase and a maximum of 20 voluntary subjects are included in the execution phase. The recruiting for the trial is exclusively carried out on a voluntary registration base through announcements at KAGES, the Medical University of Graz, the Karl-Franzens University and the University of Applied Sciences JOANNEUM, mainly focused on students and employees from the above mentioned institutions. The inclusion and exclusion criteria below are taken into consideration.

Inclusion criteria:

- sex: female / male
- age: 18-65 years
- good mental and physical capacity

Exclusion criteria:

- age: <18 years; >65 years
- pregnancy, allergy against latex or PVC, claustrophobia, hypotension (tendency to collapse), infectious diseases, affinity to thrombosis
- untreated chronic condition, for example asthma, COPD, other pulmonary diseases, cardiovascular diseases, epilepsy
- participation in another study

Methods

Each participant tested two PPE systems:

a) system A: full-body suit including respirator
  - e.g. Tychem F protective suit including socks
  - e.g. 3M™ reusable light cap S-655
  - e.g. 3M™ Jupiter™ cooling unit
  - e.g. Sempermed surgical gloves (optional)
  - e.g. boots

b) System B: full-body suit with fully integrated ventilation system (positive pressure)
  - e.g. 3M™ JS-series Typ 3 Respiratory Protective Suit (CRPS)
  - e.g. Sempermed surgical gloves (optional)
  - e.g. boots

at two different climate zones (module I: ~ 20°C / module II: ~ 28°C) on two different days. On each testing day between two and four subjects perform the series of testings listed below. Each subject is assigned through a system of randomisation called http://www.randomizer.at/, which decided with which PPE system the subject is supposed to start the testing series.

The test series described below are run in succession where the test persons start in a time-delayed fashion. After 15 minutes, they change to the next test. Between the various tests, there is a five-minute recreation break. Tests 1-3 are repeated until the test person cannot tolerate the conditions any more or stops for another reason During the test phases the following parameters are measured and recorded regularly: heart rate, heart rate variability, oxygen saturationand body temperature.
Immediately before and after the separate modules urine and saliva specimens are taken in order to create a metabolite profile, and the weight of the body and the equipment are measured in order to be able to calculate the loss of fluids.
In addition to the measurements mentioned above an extensive instruction, explanation and anamnesis will happen before the study starts. The test persons are encouraged to comment on their subjective feelings during the test series, furthermore there is an hourly survey by way of a structured questionnaire (including a continuous record on the communications system).

MODULE I a/b (environmental temperatures ~ 20°C)

**Test**	**Task**	**Type (?)**	**body position**
1	Laboratory tutorial I<br>- screwing together tubes with screwtop<br>- arranging tubes in a box following a<br>certain pattern	simulated glovebox	sitting

2	Laboratory tutorial II<br>- pipetting liquids (e.g. water) following a defined amount	simulated glovebox	standing
3	Power of concentration test	computer or handwritten	sitting

MODULE II a/b (environmental temperatures ~ 28°C)

**Test**	**task**	**Type**	**body position**
1	Laboratory tutorial I<br>- screwing together tubes with screwtop<br>- arranging tubes in a box following a<br>certain pattern	simulated glovebox	sitting
2	Laboratory tutorial II<br>- pipetting liquids (e.g. water) following a defined amount	simulated glovebox	standing
3	Power of concentration test	computer or handwritten	sitting

**Parameter**	**Measurment device**	**Unit**	**Before testing**	**During testing**	**After testing**
**Body height**	measuring tape	cm	X		
**Body weight**	personal scale	kg	X		X
**Body-Mass-Index**	calculator	BMI	X		
**Blood pressure**	blood pressure meter	mm/ Hg	X		X
**Urine**	NMR* (outsourced)		X		X
**Saliva**	NMR* (outsourced)		X		X
**Heart rate/ Variability (HR/HRV)**	heart rate monitor<br>pulse oximeter<br>ECG-monitor	beat per minut e	X	X	X
**Oxygen saturation (SpO2)**	pulse oximeter	%	X	X	X
**Core temperature**	thermometer (tba)	°C	X	X	X
**Dehydration**	personal scale	g			X

*Nuclear Magnetic Resonance (developing a metabolite profile)

Process of the study (description of a typical experimental procedure)

I PILOT PHASE

a) Recruiting of subjects

Calling for tender: MUG, KAGES, KFU, FH JOANNEUM
Generating a contact database for interested persons
Invitation for an informational event
Informational event (alternative date if required)
- presenting the trial
- conducting an informed consent discussion
- completing the case history
- distributing the informed consent

Evaluation of case histories
Collecting signed informed consents
Inclusion into the pilot phase / trial
Appointment coordination for the pilot phase
Appointment coordination for the different modules

b) Executing the pilot phase

Testing of the different PPE systems
Testing of the different laboratory tutorials / the power of concentration tests
Evaluation based on subjective judgement

c) Optimizing the study protocol based on the results of the pilot phase

II EXECUTION PHASE

a) Conducting module I a/b + II a/b

Preparation

Issuing a subject-ID
Issuing the randomly assigned PPE system
Short introduction
-> Only for the first appointment (getting dressed, explaining the testing series,…)

Initial measurements (immediately before starting the testing series)

Anamnesis (regarding the module)
Collecting saliva samples -> refrigerated storage
Collecting urine samples (total bladder emptying) -> refrigerated storage
Measuring HR (recording in the database)
Measuring oxygen saturation (recording in the database)
Measuring body temperature (recording in the database)
Measuring blood pressure (recording in the database)
Measuring body weight (without PPE system)
Measuring weight of PPE system
Installing the HR-monitor/pulse oximetry
Installing the thermometer
Getting dressed with the protective suit
Measuring the total weight (subject including the protective suit)

Conducting the testing series

Series I
Laboratory tutorial I (duration: 15 minutes + recovery period: 5 minutes)
Laboratory tutorial II (duration: 15 minutes + recovery period: 5 minutes)
Recording measured data (HR, oxygen saturation, °C)
Power of concentration test (duration: 15 minutes + recovery period: 5 minutes)
Series II
Laboratory tutorial I (duration: 15 minutes + recovery period: 5 minutes)
Laboratory tutorial II (duration: 15 minutes + recovery period: 5 minutes)
Recording measured data (HR, oxygen saturation, °C)
Power of concentration test (duration: 15 minutes + recovery period: 5 minutes)
Series III
Laboratory tutorial I (duration: 15 minutes + recovery period: 5 minutes)
Laboratory tutorial II (duration: 15 minutes + recovery period: 5 minutes)
Recording measured data (HR, oxygen saturation, °C)

Power of concentration test (duration: 15 minutes + recovery period: 5 minutes)

Series IV

Laboratory tutorial I (duration: 15 minutes + recovery period: 5 minutes)

Laboratory tutorial II (duration: 15 minutes + recovery period: 5 minutes)

Recording measured data (HR, oxygen saturation, °C)

Power of concentration test (duration: 15 minutes + recovery period: 5 minutes)

Series V (only module I)

Laboratory tutorial I (duration: 15 minutes + recovery period: 5 minutes)

Laboratory tutorial II (duration: 15 minutes + recovery period: 5 minutes)

Recording measured data (HR, oxygen saturation, °C)

Power of concentration test (duration: 15 minutes + recovery period: 5 minutes)

Series VI (only module I)

Laboratory tutorial I (duration: 15 minutes + recovery period: 5 minutes)

Laboratory tutorial II (duration: 15 minutes + recovery period: 5 minutes)

Recording measured data (HR, oxygen saturation, °C)

Power of concentration test (duration: 15 minutes + recovery period: 5 minutes)

Final measurements (immediately after finishing the testing series or after quitting the testing series)

Measuring the total weight (subject including the protective suit)

Taking off the protective suit

Measuring body weight (without PPE system)

Measuring weight of PPE system

Removing the HR-monitor/pulse oximetry

Removing the thermometer

Collecting saliva samples -> refrigerated storage

Collecting urine samples -> refrigerated storage

Measuring HR (recording in the database)

Measuring oxygen saturation (recording in the database)

Measuring body temperature (recording in the database)

Measuring blood pressure (recording in the database)

b) Data interpretation

Exporting coded subject data from Microsoft Access to Microsoft Excel

Forwarding the coded data to the statistician

Interpreting the generated data

Developing the publication

Releasing the publication to a scientific journal / dissertation

Study dropout

Each subject is able to quit the study any time (reasons are not required, but can be mentioned). Module I with an environmental temperature of about 20°C automatically ends after a maximum 6 hours. Module II with an environmental temperature of about 28°C automatically ends after a maximum of 4 hours.

Therapy safety

There is neither a benefit nor a risk for subjects included into the trial. Due to the water tightness of the protective suits used in the study strains like heat accumulation and fluid loss through heat generation inside the suit could lead to cardiovascular problems (e.g. to collapse) under certain circumstances. For this scenario a medical doctor on call-duty is contacted immediately. Apart from that, there is no further risk to be expected for the described trial.

Data management

Data management of the subject's data is exclusively made in a coded way with help of an access database, built up on the following scheme:

**Anamnesis**
subject-ID
sex
date of birth
date of anamnesis
time
body height
body weight
BMI
HR (resting)
SpO_2 in % resting
body temperature in °C resting
systolic blood pressure resting
diastolic blood pressure resting
notes

**Module I a**	**Module I b**	**Module II a**	**Module II b**
Date of examination	Date of examination	Date of examination	Date of examination
Start of testing series	Start of testing series	Start of testing series	Start of testing series
End of testing series	End of testing series	End of testing series	End of testing series
Total testing time	Total testing time	Total testing time	Total testing time
Protective suit system	Protective suit system	Protective suit system	Protective suit system
Room temperature	Room temperature	Room temperature	Room temperature
notes	notes	notes	notes
**Regular measurements during testing series**			
time	time	time	time
HR	HR	HR	HR
SpO_2	SpO_2	SpO_2	SpO_2

Body temperature ° C	Body temperature ° C	Body temperature ° C	Body temperature ° C
**Initial / final measurements**			
Systolic blood pressure	Systolic blood pressure	Systolic blood pressure	Systolic blood pressure
diastolic blood pressure	diastolic blood pressure	diastolic blood pressure	diastolic blood pressure
Body weight in g	Body weight in g	Body weight in g	Body weight in g
Weight of suit system in g	Weight of suit system in g	Weight of suit system in g	Weight of suit system in g
Total weight in g	Total weight in g	Total weight in g	Total weight in g
dehydration in g	dehydration in g	dehydration in g	dehydration in g

To guarantee a regular contact with the study subjects (for the different testing series) contact details in connection with a subject-ID are recorded in a separate, password protected database.

contact data
subject-ID
title
surname
family name
Email address
phone number
notes

Ethical aspects

Regarding benefit or risk for the subjects, see Therapy Safety above. For clarification of all ethical aspects an application to the ethical commission of the Medical University of Graz is made. All subjects are instructed at an informal meeting (repeated if required) where the informed consent paper is provided.

Data protection

Individual-related information connected with data generated within the study is exclusively saved in a coded way in a database with restricted access and own passwords. For contacting the subjects a separate contact data base with restricted access and own password is established. The generated data for analysis, publications and exchange with the collaboration partner is exclusively used in a coded way. Furthermore it is ensured that results of the trial (e.g. performance of the testing series) has, where appropriate, no influence on a current employment relationship or studies.

Estimated number of cases

The number of cases of about 20 subjects is estimated on the basis on findings of previous trials regarding testing of PPE. Due to a relatively high time requirement to go through the trial a drop out rate of about 50% is expected.

Reporting

The outcome is intended to be published in scientific journals, in a dissertation, as well as to be presented at congresses.

Literature

Bundesamt für Bevölkerungsschutz (2007). *Biologische Gefahren I, Handbuch zum Bevölkerungsschutz,* Bonn: Druckpartner Moser Druck + Verlag GmbH, Rheinbach.

(Federal Office for Civil Defence (2007). *Biological threats I, Handbook for Civil Defence*, Bonn. Publishers Moser Druck + Verlag GmbH, Rheinbach.)

Bundesamt für Bevölkerungsschutz und Katastrophenhilfe (2007). *Biologische Gefahren II. Entscheidungshilfen zu medizinisch angemessenen Vorgehensweisen in einer B-Gefahrenlage,* Bonn: Druckpartner Moser Druck + Verlag GmbH, Rheinbach.

(Federal Office for Civil Defence and Civil Protection (2007). *Biological threats II, Decision guidance on medically appropriate procedures in a B-level emergency,* Bonn. Publishers Moser Druck + Verlag GmbH, Rheinbach.)

Bundesministerium für Arbeit, Soziales und Konsumentenschutz (2009). *Arbeitsstoffe: Biologische Arbeitsstoffe (Einstufung, Schutzmaßnahmen, Branchenbeispiele),* Wien.

Federal ministry of employment, social welfare and consumer protection (2009). *Working materials: biological agents (Classification, Protective measures, Industrial examples*), Vienna.

Bundesministerium für Arbeit, Soziales und Konsumentenschutz (1998).*Verordnung der Bundesministerin für Arbeit, Gesundheit und Soziales über de Schutz der Arbeitnehmer/innen gegen Gefährdung durch biologische Arbeitsstoffe (Verordnung biologische Arbeitsstoffe - VbA),* Wien.

Federal ministry of employment, social welfare and consumer protection (1998). *Regulation of the federal ministry of employment, health and social welfare on protection of employees from hazards from working materials (Regulation of biological agents)*, Vienna.

Fleming, D.O. & Hunt, D.L. (2006). *Biological Safety, Principles and Practices,* 4th Edition, Washington DC: ASM Press.

Li, L et al. (2005). Biosafety level 3 laboratory for autopsies of patients with severe acute respiratory syndrome: principles, practices, and prospects. Clinical infectious diseases : an official publication of the Infectious Diseases Society of America, 41(6), pp. 815-821.

Marklund, L.A. (2003). Patient care in a biological safety level-4 (BSL-4) environment. Critical care nursing clinics of North America, 15(2), pp. 245-255.

Annex

Proposal to the ethics commission
Information for study population / declaration of consent
Proof of qualification of auditor
Declaration of conflict of interests
Call for tender
Compensation for testing persons

Signature:……………………………….
(Auditor)
