# Supplementary material for: Limiting factors for wearing personal protective equipment (PPE) in a health care environment evaluated in a randomised study
Source: PLoS One. 2019 Jan 22;14(1):e0210775. doi: 10.1371/journal.pone.0210775 (PMC6342303; doi:10.1371/journal.pone.0210775)
Supplement: S3 File — (PDF) [file pone.0210775.s003.pdf]

Amendment Nr: 1 zum Studienprotokoll Version 1.2 / 15.04.2011
Titel: Testung von persönlicher Schutzausrüstung (PSA) für ein BSL4 Umfeld
EK Nr.: 23-321 ex 10/11, Datum des Votums: 21.04.2011

Studiendesign

Bei dieser Studie handelt es sich um eine monozentrisch durchgeführte, randomisierte, cross-over Studie. Im Rahmen der Studie wird von jedem Probanden eines der beiden Schutzanzugssysteme (Zuteilung wird randomisiert), bei jeweils zwei unterschiedlichen Temperaturbedingungen, gestestet und die Ergebnisse gegenübergestellt. Diese Studie dient zur Ermittlung der körperlichen Leistungsfähigkeit, der Konzentrations-/Reaktionsfähigkeit, von Fehlerraten und des subjektiven Befindens bei Laborarbeiten mit persönlicher Schutzausrüstung. Es ist eine Pilotphase geplant, die zur Optimierung der simulierten Laborarbeit in Gloveboxen, zur Definition möglichst aussagekräftiger Simulationsbedingungen (z.B. wieviele Pipettierschritte in einer bestimmten Zeit machbar sind, Testung der Logistik während der Durchführung), sowie zur Ermittlung der Relevanz der geplanten Messungen dienen soll. In die Pilotphase werden zwischen 3-5 freiwillige Probanden eingeschlossen. Basierend auf Erkenntnissen publizierter Studien, in Bezug auf Testung von persönlicher Schutzausrüstung, wird von einer Probandenzahl von maximal 20 Personen ausgegangen, wobei aufgrund des relativ hohen Zeitaufwandes für die Studie mit einer Dropout-Rate von ca. 50% gerechnet werden muss.

Methoden

Alle Studienteilnehmer testen eines der beiden Schutzanzugssysteme:

a) System A: Ganzkörperoverall mit Respirator
   - z.B. Tychem F Schutzanzug inkl. Socken
   - z.B. 3M™ Mehrwegleichthaube S-655
   - z.B. 3M™ Jupiter™ Gebläseeinheit
   - z.B. Sempermed OP-Handschuhe (optional)
   - z.B. Stiefeln

b) System B: Ganzkörperanzug mit ingegriertem Belüftungssyystem (Überdruck)
   - z.B. 3M™ JS-Serie Typ 3 Respiratory Protective Suit (CRPS)
   - z.B. Sempermed OP-Handschuhe (optional)
   - z.B. Stiefeln

in zwei unterschiedlichen Temperaturkategorien (Kategorie I: ~ 20°C / Kategorie II: ~ 28°C) zu insgesamt zwei Terminen. Zu jedem Termin führen zwischen zwei und vier Proband/Innen die unten angeführten Testreihen durch. Die Zuteilung mit welchem Schutzanzugsystem der/die Proband/in in jeder Temperaturkategorie alle Testungen durchführt erfolgt über ein Randomisierungstool: http://www.randomizer.at/.

Die unten angeführten Testreihen laufen hintereinander ab, wobei die Proband/Innen zeitversetzt beginnen. Nach 15 Minuten wird zum nächsten Test gewechselt. Zwischen den einzelnen Testungen erfolgt eine 5-minütige Erholungspause. Die Tests 1-4 werden so lange wiederholt, so lange der/die Proband/In die gegebenen Bedingungen toleriert oder bis er aus anderen Gründen abbricht (Temperaturkategorie I: max. 6 Std. / Temperaturkategorie II: max. 4 Std.). Während der Testphasen werden Herzfrequenz (HF) / Herzfrequenzvariabilität (HFV) und Körperkerntemperatur gemessen und in regelmäßigen Abständen aufgezeichnet. Unmittelbar vor und nach der Absolvierung der einzelnen Testkategorien werden Urin- und Speichelproben, zur Erstellung eines Metabolitenprofils entnommen, sowie Körpergewicht und Gewicht des Anzugsystems, zur späteren Berechnung des Flüssigkeitsverlustes, gemessen. Zusätzlich zu den genannten Messungen erfolgt eine ausführliche Einschulung, Aufklärung und Anamnese vor Beginn der Studie. Die Proband/Innen werden aufgefordert sich über ihr subjektives Befinden während der Durchführung der Testreihen zu äußern, zudem erfolgt eine stündliche Befragung mittels strukturiertem Fragebogen (es erfolgt eine durchgehende Aufzeichnung über das Kommunikationssystem).

Temperaturkategorie I A/B (Umgebungstemperatur ~ 20°C)

Test	Tätigkeit	Art	Position
1	Laborübung I<br>- Probenröhrchen zusammenschrauben<br>- nach vorgegebenem Muster einordnen	simulierte Glovebox	sitzend
2	Laborübung II<br>- Flüssigkeit (z.B. Wasser) pipettieren<br>- nach vorgegebenen Mengen	simulierte Glovebox	stehend
3	Konzentrationstest	am PC od. schriftlich	sitzend
4	Reaktionstest	am PC	stehend

Temperaturkategorie II A/B (Umgebungstemperatur ~ 28°C)

Test	Tätigkeit	Art	Position
1	Laborübung I<br>- Probenröhrchen zusammenschrauben<br>- nach vorgegebenem Muster einordnen	simulierte Glovebox	sitzend
2	Laborübung II<br>- Flüssigkeit (z.B. Wasser) pipettieren<br>- nach vorgegebenen Mengen	simulierte Glovebox	stehend
3	Konzentrationstest	am PC od. schriftlich	sitzend
4	Reaktionstest	am PC	stehend
