# Supplementary material for: Limiting factors for wearing personal protective equipment (PPE) in a health care environment evaluated in a randomised study
Source: PLoS One. 2019 Jan 22;14(1):e0210775. doi: 10.1371/journal.pone.0210775 (PMC6342303; doi:10.1371/journal.pone.0210775)
Supplement: S4 File — (PDF) [file pone.0210775.s004.pdf]

Amendment Nr: 1 of Study Protocoll Version 1.2 / 15.04.2011
Title: Testing personal protective equipment (PPE) for a BSL4 Environment
EK Nr.: 23-321 ex 10/11, Date of Vote: 21.04.2011

Study Design

This study is planned as a monocentric, randomised cross-over study. During the course of the study, one of two protective suits will be assigned to each study participant (randomised assignment), tested under two different temperature conditions, and the results compared. This study serves to determine the bodily performance, the concentration /reactivity levels, error rates, and subjective well being during laboratory work in protective suits. A pilot phase is planned, in order to optimise the simulated laboratory work in glove boxes, to define the most meaningful simulated conditions (e.g., how many pipetting steps are possible in a given time, testing the logistics during the procedure), as well as to determine the relevance of the planned measurements. In the pilot phase, between 3-5 voluntary test subjects will be included. Based on the evidence from published studies regarding testing of personal protective equipment, a participant number of maximally 20 is assumed, although, due to the relatively long time required for the study, a dropout rate of about 50% is to be expected.

Methods

All study participants test one of two protective suits.

a) System A: Whole body overall with respirator
   - e.g., Tychem F protective suit incl. socks
   - e.g., 3M™ Hood S-655
   - e.g., 3M™ Jupiter™ Powered Air Turbo
   - e.g., Sempermed OP-gloves (optional)
   - e.g., Boots

b) System B: Whole body suit with integrated ventilation system (overpressure)
   - e.g., 3M™ JS-Serie Type 3 Respiratory Protective Suit (CRPS)
   - e.g., Sempermed OP-gloves (optional)
   - e.g., Boots

in two different temperature categories (category I: ~ 20°C / category II: ~ 28°C) in two different sessions. In each session, between two and four test subjects carry out the test series listed below. The assignment of which suit is to be worn by the test subject for performing all the tests in each temperature category is decided using a randomisation tool: http://www.randomizer.at/.

The test series listed below run sequentially, with the test subjects starting in a time-lagged manner. After 15 minutes, the test subject switches to the next test. Between the individual tests, there is a 5 minute recovery break. The tests 1-4 are repeated as long as the test subject tolerates the conditions or until he/she terminates due to other reasons (temperature category I: max. 6 hours / temperature category II: max. 4 hours). During the test phase, heart rate, heart rate variability and body temperature are measured and recorded in regular intervals. Immediately before and after the individual test categories, urine and saliva samples are taken to enable construction of a metabolite profile, and body weight and weight of the suit are measured, in order to later calculate the loss of fluids. In addition to these measurements, comprehensive instruction and anamnesis are carried out prior to commencement of the study. The test subjects are instructed to speak out about their subjective well being during performance of the test series. In addition, there is an hourly survey conducted using a structured questionnaire (continuous recording via the communication system).

Temperature Category I A/B (ambient temperature ~ 20°C)

Test	Activity	Type	Position
1	Laboratory exercise I<br>- screw caps on sample tubes<br>- order according to a given pattern	simulated Glovebox	sitting
2	Laboratory exercise II<br>- pipette liquid (e.g., water)<br>- according to given amounts	simulated Glovebox	standing
3	Concentration test	on PC or written	sitting
4	Reaction test	on PC	standing

Temperature Category II A/B (ambient temperature ~ 28°C)

Test	Activity	Type	Position
1	Laboratory exercise I<br>- screw caps on sample tubes<br>- order according to a given pattern	simulated Glovebox	sitting
2	Laboratory exercise II<br>- pipette liquid (e.g., water)<br>- according to given amounts	simulated Glovebox	standing
3	Concentration test	on PC or written	sitting
4	Reaction test	on PC	standing
